# Characterization of the Central Sulcus *Pli‐De‐Passage Fronto‐Pariétal Moyen* in > 1000 Human Brains

**DOI:** 10.1002/hbm.70457

**Published:** 2026-01-30

**Authors:** Anna Marie Muellen, Renate Schweizer

**Affiliations:** ^1^ University of Goettingen Goettingen Germany; ^2^ Functional Imaging Laboratory German Primate Center Goettingen Germany; ^3^ Cognitive Neuroscience Laboratory German Primate Center Goettingen Germany; ^4^ Leibniz Science Campus Primate Cognition Goettingen Germany

**Keywords:** central sulcus, human neuroanatomy, structural magnetic resonance imaging

## Abstract

The *pli‐de‐passage fronto‐pariétal moyen* (PPfpm), a deep cerebral fold of the human brain, presents as a common though small elevation at the central sulcus (CS) fundus where it connects the pre‐ and postcentral gyri at the level of the sensorimotor hand area. Given the PPfpm's location, single case‐reports of its association with the functional sensorimotor hand area, and evidence linking it to the somato‐cognitive action network, it holds potential as an anatomical landmark for the sensorimotor region. To characterize the macroscopic morphology of the PPfpm and evaluate its relevance as a reliable and easily detectable anatomical landmark, methods for observer‐independent characterization of cortical sulci and structures were adapted and developed to investigate the PPfpm in a large dataset. For 1112 subjects from the Human Connectome Project Young Adult S1200 Release, CS depth profiles were computed from structural magnetic resonance imaging (MRI) data, and an algorithm was developed to automatically extract the PPfpm from these depth profiles. Based on the extraction of two key features approximating the PPfpm at its peak height (PPfpm‐I) and its lateral end (PPfpm‐II), a principal description of the PPfpm's position and extent as influenced by hemisphere, handedness, and sex was conducted. Analyses revealed the PPfpm as a near‐universal cerebral fold in the adult human brain, consistently located at mid‐height within the CS with a strong association to the CS sulcal pits. Though commonly of small extent, the PPfpm can be reliably identified in CS depth profiles and in structural MRI data. By providing a systematic, modern macroanatomical characterization of the PPfpm in a large cohort with rigorous quality control, the present study demonstrates the potential of the PPfpm to serve as a robust anatomical landmark for the sensorimotor hand and digit area of the human brain.

## Graphical abstract

The *pli‐de‐passage fronto‐pariétal moyen* (PPfpm), a deep fold within the central sulcus (CS), was characterized in 2165 MRI‐derived CS depth profiles. Realignment to their deepest points enabled automated PPfpm extraction at its height (PPfpm‐I) and lateral end (PPfpm‐II). Anatomically validated quality control confirmed the PPfpm as a robust sensorimotor landmark.

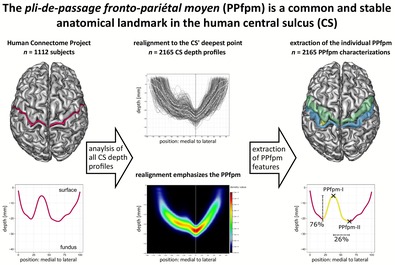

## 1 Introduction

First described by Broca (1888), the three *pli‐de‐passages* of the human central sulcus (CS) are distinct cerebral folds that connect the precentral gyrus of the frontal lobe and the postcentral gyrus of the parietal lobe by traversing the CS—a primary fissure that typically extends continuously from the interhemispheric cleft to the lateral sulcus. Among these *pli‐de‐passages*, the *pli‐de‐passage fronto‐pariétal supérieur* (PPfps) and the *pli‐de‐passage fronto‐pariétal inférieur* (PPfpi) are prominent, superficial connections that demarcate the superomedial and inferolateral boundaries of the CS. The PPfps is thereby located within the paracentral lobule, while the PPfpi corresponds—in modern terminology—to the subcentral gyrus. While integral to the broader concept of the CS *pli‐de‐passages*, the PPfps and PPfpi are not the focus of the present study. Rather, this work aims to describe and characterize in detail the third and less well‐known structure: the *pli‐de‐passage fronto‐pariétal moyen*.

### 1.1 Anatomical Studies Relating to the *Pli‐de‐Passage Fronto‐Pariétal Moyen*

Described as a deep fold (“*pli‐*”) of small extent (“toujours très profond”) passing (“*de‐passage*”) from frontal to parietal lobe (“*fronto‐pariétal*”) in the middle/at mid‐height (“*moyen*”) of the CS (Broca 1888), the *pli‐de‐passage fronto‐pariétal moyen* (PPfpm) comprises a small and hidden cerebral fold at the fundus of the CS. The PPfpm is shown in a single report (Cunningham 1890) to arise during intrauterine brain development where it presents as an “eminence” that initially separates the CS into a shorter upper (superior genu of the CS, ca. 1/3) and a longer lower piece (inferior genu of the CS, ca. 2/3). Parallel to the acceleration of cortical growth around the 5th month of intrauterine brain development and in parallel with the development of the CS between the 20th and 23rd gestational week (Hostalet et al. 2025; Wagner 1860; White et al. 2010), it is progressively displaced toward the CS fundus while the two CS pieces fuse above it to one continuous fissure (Cunningham 1890). In the adult brain, the PPfpm persists as a remnant of fetal morphology (Cunningham 1890), appearing as a small elevation (Cykowski et al. 2008) or a deep annectant gyrus/cerebral fold (“*Tiefenwindung*”) at the CS fundus at its superomedial third (Eberstaller 1890; Heschl 1877; Schweizer et al. 2025).

Due to its concealed location, the PPfpm is less known, and its height has been characterized in only one large historic dataset from 1877 (Heschl 1877), a study recently replicated by Schweizer et al. (2025). These investigations confirmed that, while generally of small height < ⅙ of CS depth, the PPfpm may occasionally remain superficial in the adult brain, thus discontinuing the CS and separating it in its original upper and lower piece. Previously described only in single findings (Alkadhi and Kollias 2004; Cunningham 1890; Ecker 1869; Féré 1891; McKay et al. 2013; Schweizer et al. 2014; Turner 1866; Wagner 1862), two recent studies confirmed the low prevalence of the superficial PPfpm in large cohorts as < 1% (Mangin et al. 2019; Muellen and Schweizer 2022; Schweizer et al. 2019, 2025). To date, there is no evidence suggesting any pathological significance of this mere anatomical variation.

The anatomy of the CS *pli‐de‐passages* as cerebral folds traversing the CS was recently confirmed by Skandalakis et al. (2025) through white matter microdissections of 16 human cadaveric brains. Here, the *pli‐de‐passages* are described as short connections consisting of grey and primarily white matter. The PPfpm, specifically, was generally located at the level of the middle frontal gyrus, consistent with historical descriptions placing it at the superomedial third of the CS. Studies on the PPfpm in the broader context of CS morphology (Cykowski et al. 2008) in 55 subjects support this finding, confirming that, while the PPfpm is morphologically variable, it remains positionally stable and is generally of a small height. Notably, the PPfpm is further implied to be flanked by the sulcal pits of the CS (Im et al. 2010; McKay et al. 2013), developmentally early and spatially stable landmarks within cortical sulci (Hostalet et al. 2025; Lohmann et al. 2008; Régis et al. 2005), suggesting a link between the PPfpm and underlying cortical organization.

Elucidating the microanatomical cortical organization relevant to the PPfpm, it is reasonable to assume that—given its position at the CS fundus—the PPfpm lies near the cytoarchitectonic border between Brodmann area (BA) 4 and BA3 (Geyer et al. 1999, 2000; White et al. 1997a). Recent work similarly links the PPfpm to this transition zone (Skandalakis et al. 2025). However, the considerable variability in the BA4/BA3 border (Geyer et al. 1999, 2000; Rademacher et al. 2001; White et al. 1997a), together with the scarcity of cytoarchitectonic studies specifically examining the PPfpm, leaves its detailed microanatomical composition largely unresolved to date.

Taken together, historical and contemporary studies describe the PPfpm as a common structure within the CS of the human brain, characterized by its small extent and only rare superficial variations, its consistent location at the superomedial third of the CS, and its association with the CS sulcal pits. Despite this apparent anatomical consistency, the precise position and extent of the PPfpm have, however, not been systematically characterized in a large cohort. Thus, fundamental questions regarding its precise morphology and the degree of its macroanatomical variability across individuals have not been addressed.

### 1.2 Association of the *Pli‐de‐Passage Fronto‐Pariétal Moyen* With Functional Sensorimotor Areas

As described above, the PPfpm is located at the superomedial third of the CS, where it connects the precentral gyrus containing the primary motor cortex (M1) and the postcentral gyrus containing the primary somatosensory cortex (S1) (e.g., Penfield and Boldrey 1937; Penfield and Rasmussen 1950; Saadon‐Grosman et al. 2020) at the level of the functional sensorimotor hand/digit area.

Situated near the “hand knob”, a prominent knob‐like folding of the precentral gyrus associated with the functional M1 hand area (Caulo et al. 2007; Rodrigues et al. 2015; Rumeau et al. 1994; Ten Donkelaar et al. 2018; Wagner et al. 2013; Yousry 1997), the PPfpm may also be associated with sensorimotor hand function. Supporting this, in a rare individual case of a superficial PPfpm discontinuing the CS, the PPfpm was shown to relate to the functional M1 hand area (Alkadhi and Kollias 2004) during a blood‐oxygenation‐level‐dependent functional MRI study. Following finger‐to‐thumb opposition of the dominant right hand, wrist flexion and extension, and elbow flexion and extension, the superficial PPfpm of this individual was associated with the M1 hand area and described as separating it from the functional elbow representation. Additionally, in a combined structural and functional MRI study, Germann et al. (2020) reported a close relationship between CS morphology and sensorimotor representations following a set of 14 motor tasks. The PPfpm, herein described as a “submerged gyrus”, was found to lie between two distinct CS segments, separating the sensorimotor representations of the fingers from those of the facial areas. Further evidence of the PPfpm's association with functional specialization arises from recent work by Gordon et al. (2023) and Skandalakis et al. (2025). Gordon et al. (2023) thereby challenged the classical somatotopic organizational principle of M1 and proposed the “integrate‐isolate model”. Within this framework, three effector regions—specific for regional movements—alternate with three inter‐effector regions—involved in whole‐body action—forming the somato‐cognitive action network (SCAN). Given SCAN's distinct spatial pattern along the precentral gyrus, Skandalakis et al. (2025) proposed that the CS *pli‐de‐passages* may serve as its neurobiological correlates. The PPfpm thereby corresponds to the middle inter‐effector region between the hand and face effector region, highlighting its potential as an anatomical landmark for SCAN and its close spatial relationship with the M1 hand area to which it lies directly lateral. Similarly, Jensen et al. (2023) recently described the “Rolandic motor association” area as a region in the depth of the CS that was observed in 13 subjects to be related to movement coordination. While not explicitly stated and not described with respect to the CS *pli‐de‐passages*, the location of this area as presented on structural MRI data strongly suggests a link to the PPfpm.

The PPfpm may also relate to the functional S1 hand area: In a clinical Positron‐Emission‐Tomography study during the presurgical evaluation of 27 tumor and epilepsy patients, Boling and Olivier (2004) showed that peak activation of the functional S1 hand area—elicited through vibrotactile stimulation of the whole hand using a handheld vibrating ball—was located reliably at the PPfpm.

Thus, an increasing body of evidence suggests a close association between the PPfpm and the functional sensorimotor hand/digit area. However, the absence of a detailed anatomical characterization of the PPfpm, along with its limited mention in the existing literature, continues to hinder its broader utilization as an anatomical landmark.

### 1.3 Aim of the Present Study

A comprehensive characterization of the PPfpm's macroanatomy is essential to establish it as a robust anatomical landmark within the central region. Despite its recognized relevance, detailed data on the morphology and interindividual variability of the PPfpm, including the influence of key biological factors, remain sparse. The present study addresses this gap through a systematic, large‐scale analysis of the PPfpm's macroanatomy with rigorous quality control. Consequently, this study aims to enable the reliable identification of the PPfpm, thereby promoting its application as a robust anatomical landmark in both research and clinical practice.

Informed by an established framework for an observer‐independent characterization of cortical sulci (Cykowski et al. 2008), a novel method was implemented to extract the PPfpm from structural MRI data in a large cohort of 1112 subjects of the Human Connectome Project Young Adult (HCP‐YA) S1200 Release. Following extraction, the macroanatomical extent and position of the PPfpm as influenced by hemispheric location and the subjects' handedness and sex were characterized. Lastly, findings were contextualized within the framework of current literature.

## 2 Materials and Methods

### 2.1 Subjects and Magnetic Resonance Imaging Data

For 1112 subjects (506 males, 606 females; age in years: 28.80 ± 3.70, range 22–37) of the HCP‐YA (Van Essen et al. 2013) S1200 Release (release date: 01 March 2017), unprocessed structural whole‐brain T1‐weighted MRI data were downloaded. All data were obtained on a 3 Tesla MRI scanner (Connectome Skyra, Siemens, Erlangen) with a 32‐channel head coil, and a 3D MPRAGE sequence (TR = 2400 ms; TE = 2.14 ms; TI = 1000 ms; flip angle 8°; iPAT = 2, FOV = 224 × 224 mm^2^; spatial resolution = 0.7 × 0.7 × 0.7 mm^3^; TA = 7:40 min).

### 2.2 Image Preprocessing, Manual Selection and Sulcal Mesh Parametrization of the Central Sulcus

**FIGURE 1:**
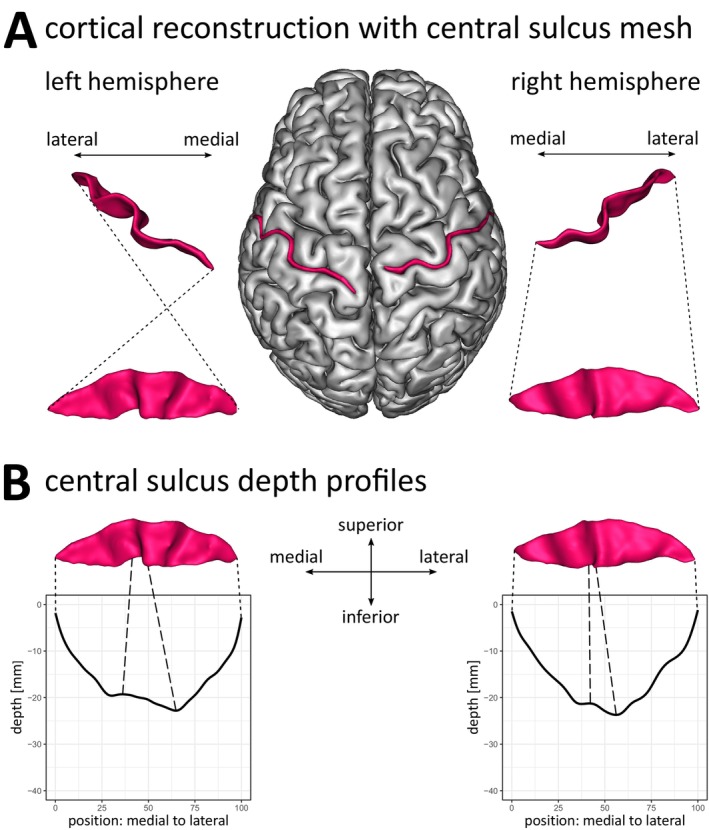
Relation between reconstructed central sulcus meshes and depth profiles. (A) Grey matter surface reconstruction and reconstructed CS mesh (red) of an exemplary subject. (B) Relation between the CS meshes of the left and right hemisphere with their respective depth profiles. Depth values are given at 101 discrete, equidistant positions from the medial (position: 0) to the lateral end of the CS (position: 100). Depth values were inverted resulting in a depth profile starting at 0 and going into negative values with increasing depth. Dotted lines mark the equivalent medial and lateral end of the reconstructed CS mesh and the depth profile. Dashed lines mark the *pli‐de‐passage fronto‐pariétal moyen* within the CS mesh and the equivalent point in the depth profile.

All data were analyzed with BrainVISA (BrainVISA 4.5.0; Morphologist 2015 Pipeline; https://brainvisa.info/). Image preprocessing included manual selection of anterior (AC) and posterior commissure (PC), reorientation to AC‐PC‐plane and inhomogeneity correction. White and grey matter surface reconstructions were obtained for each hemisphere separately. Within the Morphologist 2015 Pipeline, cortical fold graphs (CFGs) constituting a set of attributed relational graph structures following the medium plane of all sulci (Mangin et al. 2004; Régis et al. 2005) were created for each hemisphere of all subjects. For each hemisphere, the CFGs of the entire hemisphere were visually inspected to identify the CS. An individual CS consists of usually two CFGs (range one to five CFGs), with each single CFG being limited by the anatomical extremities of the sulci—the brain surface and the fundus of the sulci—and constrained by side branches and deep cerebral folds buried within the sulci (Régis et al. 2005). To ensure a correct and complete identification of the CS, all CFGs of each individual CS were selected manually and parametrized with BrainVISA's Sulcus Parametrization function. The resulting hemispheric datasets consist of a rendered CS mesh (Figure 1A) and its depth profile (Figure 1B) for each examined hemisphere. The CS depth profile thereby approximates true CS depth based on the reconstructed CS mesh along its medial to lateral trajectory in a set of 101 discrete data points iϵ1:101, with the medial end at position $x_{1}=0$ near the interhemispheric cleft and the lateral end near the lateral sulcus at $x_{101}=100$. At each of the 101 positions, the depth values $y_{i}$ are computed as length in mm from the top ridge of the CS mesh to its fundus (Cykowski et al. 2008).

### 2.3 Exclusion of Data and Quality Control

Of the originally obtained 2224 hemispheric datasets from 1112 subjects, 59 hemispheric datasets from 56 subjects were excluded from all further analysis. In 53 subjects, either the left (29 subjects) or the right hemisphere (24 subjects), and for three subjects both the left and right hemispheric datasets were omitted. Reasons for exclusion (multiple can apply to one dataset) included: data loss during download and computations (5 datasets), incorrect computation of CFGs (29 datasets), incorrect manual selection of CS parts (16 datasets), and anatomical anomalies of the CS which prevented correct computations and selection (30 datasets). Anatomical anomalies included nine cases of a discontinuous CS caused by a superficial PPfpm at brain surface levels (Schweizer et al. 2025). All analyses were performed on the remaining 2165 hemispheric datasets from 1109 subjects (see Table 1, Results).

**TABLE 1:** Hemispheric datasets after quality control of data.

Subjects						Hemispheres		
Groups	Brains (*n*)	Age				Datasets (*n*)		
		M ± SD	SEM	Min	Max	Total	Right	Left
Total	1109	28.80 ± 3.70	0.11	22	37	2165	1085	1080
Male	503	27.89 ± 3.62	0.16	22	37	979	491	488
Female	606	29.56 ± 3.60	0.15	22	36	1186	594	592

Abbreviations: M, mean; SD, standard deviation; SEM, standard error of the mean.

### 2.4 Analysis of Depth Profiles and Identification of Stable Features for the *Pli‐de‐Passage Fronto‐Pariétal Moyen*

Analyses of 2165 left and right hemispheric depth profiles were performed with the software R (R 3.6.1 and R 4.2.2; https://www.R‐project.org/). For analysis and graphical representation of depth profiles, the depth values $y_{i}$ were inverted to result in a depth profile starting at 0 and going into negative values with increasing depth. All depth profiles were smoothed with a cubic smoothing spline, effectively applying a low‐pass filter to suppress high‐frequency noise arising from the discrete measurements, while preserving the depth profiles' trajectories (package: stats; function: smooth. spline; generalized cross validation with coefficient of penalty 1, knots defined as all positions $x_{i}$; degrees of freedom 20).

All extrema of the 2165 depth profiles, that is, all local and global maxima and minima, were extracted (package: pastecs; function: turnpoints; Grosjean and Ibanez 2018). A two‐dimensional kernel density estimation of depth values and positions was computed for all maxima (*n* = 3310), all minima (*n* = 5475), and all global minima (*n* = 2165) to extract clusters of maxima and minima across all depth profiles. Clusters were evaluated on a square grid (100 × 100) defined by each the outermost values (package: MASS; function: kde2d; Ripley and Venables 2019).

To extract the PPfpm of individual depth profiles, the global minimum at positions > 45 was defined and computed as the feature “PPfpm‐II”, marking the lateral end of the PPfpm. A second feature, “PPfpm‐I”, was chosen in relation to PPfpm‐II as the next medial local maximum to describe the PPfpm at its height. In the infrequent case of a double elevation of the PPfpm, PPfpm‐I is thus defined to coherently identify the height of the PPfpm at the directly adjacent maximum to PPfpm‐II.

### 2.5 Quality Control of the Identified Features PPfpm‐I and PPfpm‐II

Quality was manually assessed for all 2165 hemispheric datasets by one of the authors (AMM) for a thorough and specific validation of the applied method: Assessment of PPfpm‐I and PPfpm‐II was performed by visual inspection based on a graphical representation of depth profiles with marked PPfpm‐I/‐II. Depth profiles with clearly correct features (e.g., a single PPfpm elevation with correct placement of PPfpm‐I as peak height and PPfpm‐II as lateral end) were marked as correct, whereas obviously incorrect features (e.g., misalignment of PPfpm‐II as the medial end of a single PPfpm elevation) were marked as incorrect. For depth profiles with an ambiguous PPfpm shape or uncertain features (e.g., a double or indistinct PPfpm elevation), the respective reconstructed CS mesh was inspected: extracted PPfpm‐I/‐II were visually matched to their anatomical counterparts on the CS mesh. For all hemispheric datasets where anatomical structures could not be conclusively inferred from depth profiles and CS meshes alone, cortical white and grey matter reconstructions and structural MRI data were included in the assessment. The hemispheric datasets were labeled “correct” when PPfpm‐I and PPfpm‐II aligned and confirmed clearly with their anatomical equivalent. Depth profiles with at least one feature not aligning with its anatomical equivalent, whether due to anatomical anomalies or failures of the method, were labeled as “incorrect”. Inconclusive hemispheric datasets were marked as “incorrect”. To ensure consistency during the confirmation procedure, previously labeled hemispheric datasets were reassessed in regular intervals. A divergence rate of more than 5% led to re‐evaluation of all respective hemispheric datasets. Interim results on the correctness rates were blinded until the validation procedure was finalized.

### 2.6 Height and Partial Width of the *Pli‐de‐Passage Fronto‐* *P* *ariétal Moyen*

The absolute height of the PPfpm (PPfpm absolute height) was calculated for all data with both PPfpm‐I and PPfpm‐II extracted (*n* = 1983, 91.60%), and defined as their depth difference. The relative height (PPfpm relative height) was defined as the ratio of absolute height to depth of PPfpm‐II. Since PPfpm‐II coincides in most cases (*n* = 2079, 96.03%) with the overall deepest point of the CS, this equals a normalization procedure of absolute PPfpm height to maximal CS depth. The width of the PPfpm was computed as a partial width (PPfpm peak‐to‐lateral width) between the PPfpm's peak height (PPfpm‐I) and its lateral end (PPfpm‐II). It is defined as the absolute difference of positional values of PPfpm‐I and PPfpm‐II and—given the normalization of CS length to positional values 0 to 100—the PPfpm peak‐to‐lateral width is expressed as a percentage of total CS length.

$$\text{PPfpm absolute height}=|y_{i}\;\left(\text{PPfpm}-\mathrm{II}\right)-y_{i}\left(\text{PPfpm}-I\right)|$$

$$\text{PPfpm relative height}=|\frac{y_{i}\;\left(\text{PPfpm}-\mathrm{II}\right)-y_{i}\left(\text{PPfpm}-I\right)}{y_{i}\;\left(\text{PPfpm}-\mathrm{II}\right)}|$$

$$\text{PPfpm peak}-\text{to}-\text{lateral width}=|x_{i}\;\left(\text{PPfpm}-\mathrm{II}\right)-x_{i}\left(\text{PPfpm}-I\right)|$$

### 2.7 Statistical Analysis of the *Pli‐de‐Passage Fronto‐* *P* *ariétal Moyen*

All statistical analyses were performed on a subset of 1556 hemispheric datasets from 778 subjects (male: *n* = 374, female: *n* = 404) where both hemispheric datasets exhibit correctly extracted PPfpm‐I/‐II (Table 3). Descriptive statistics of PPfpm‐I/‐II values in this subset showed no notable deviation from those of the complete sample.

Three‐way mixed‐effects model analysis of variance (ANOVA) (package: ez; function: ezANOVA; type 3 Sum of Squares; Lawrence 2016) was computed to analyze the effect of “feature” (within‐subject factor: PPfpm‐I, PPfpm‐II), “hemisphere” (within‐subject factor: right, left), and “sex” (between‐subject factor: male, female; given by the HCP‐YA as gender, and defined as the stated biological sex of subjects) on position and, separately, on depth of PPfpm‐I and PPfpm‐II. Normality of distribution for all groups (Shapiro–Wilk, quantile‐quantile plots), and homoscedasticity of variance (Levene; cross design on mean for “sex” and “hemisphere” per “feature”) were reviewed. The variable “sex” was not balanced across groups. For all ANOVA effects significant at *p* < 0.05, post hoc two‐sided paired *t*‐tests were performed with appropriate Bonferroni correction of the significance threshold *p*_crit_.

Additional statistical analyses were conducted to examine potential effects of handedness on the features PPfpm‐I and PPfpm‐II. In the HCP‐YA, handedness is provided as the Oldfield Index from the Edinburgh Handedness Inventory (Oldfield 1971), ranging from −100 (left‐handed) to +100 (right‐handed) in 5‐point increments. In the present analysis, left‐handedness was defined as Oldfield Index < 0 (*n* = 67 subjects) and right‐handedness as Oldfield Index > 0 (*n* = 708 subjects); subjects with an index of 0 were excluded to avoid ambiguity (*n* = 6 subjects). Given the pronounced imbalance between left‐ and right‐handed subjects, and the skewed distribution of the Oldfield Index toward strong right‐handedness at +100, a threefold statistical approach was implemented to ensure robustness.

First, a balanced resampling (“bootstrap‐like”) procedure was applied within a mixed‐effects model ANOVA to equalize left‐ and right‐handedness across mirrored Oldfield Indices. This bootstrap‐like mixed‐effects model ANOVA was complemented by (A) a permutation analysis in which handedness labels were randomly reassigned across subjects to compute empirical *p*‐values, and (B) a sensitivity analysis using a linear mixed‐effects model.

A three‐way mixed‐effects model ANOVA was implemented with “feature” (within‐subject factor: PPfpm‐I, PPfpm‐II), “hemisphere” (within‐subject factor: left, right), and “handedness” (between‐subject factor: left‐handed, right‐handed) as predictors for position and, separately, depth of features. To correct for unequal group sizes and asymmetric distribution of Oldfield Indices, all left‐handed subjects were matched to right‐handed subjects with mirrored Oldfield Indices. A bootstrap‐like balanced resampling (*n* = 10.000 iterations) was applied, with the ANOVA recomputed for each iteration.

A complementary permutation analysis (*n* = 10.000 iterations) was conducted to obtain empirical, model‐free significance estimates. “Handedness” (left‐handed, right‐handed) was randomly reassigned across subjects, while maintaining original group sizes. For each permutation, the mixed‐effects model ANOVA was recomputed for position and, separately, depth of features. Empirical *p*‐values were derived according to Phipson and Smyth (2010), and evaluated at significance *p* < 0.05.

A complementary linear mixed‐effects model (restricted maximum likelihood) was implemented to assess the robustness of results with respect to within‐subject variability (package: lme4; function: lmer; version: 1.1.30; Bates et al. 2015). The model included “feature” (PPfpm‐I, PPfpm‐II), “hemisphere” (left, right), and “handedness” (left‐handed, right‐handed) as fixed effects with all possible interactions. A random intercept per subject was included to account for repeated measurements across hemispheres and features. Significance was evaluated based on model‐estimated *t* statistics. Separate models were computed for position and depth of features.

Three‐way mixed‐effects model ANOVA was conducted to study the effects of “dimension” (within‐subject factor: PPfpm peak‐to‐lateral width, PPfpm relative height), “hemisphere” (within‐subject factor: left, right), and “sex” (between‐subject factor: male, female) to characterize the PPfpm's extent further. Supplementary two‐way mixed‐effects model ANOVAs were performed on each the PPfpm peak‐to‐lateral width, the relative PPfpm height, and on the absolute PPfpm height to closer investigate the observed effects of “hemisphere” (within‐subject factor: left, right) and “sex” (between‐subject factor: male, female). For all analyses, appropriate review of normality of distribution (Shapiro–Wilk, quantile‐quantile plots) and homoscedasticity of variance (Levene; cross design on mean for sex and hemisphere per type of dimension) was performed.

Pearson product–moment correlation coefficient was computed to investigate potential interdependence and potential effects of hemispheric origin on PPfpm peak‐to‐lateral width and relative PPfpm height.

## 3 Results

### 3.1 The *Pli‐de‐Passage Fronto‐Pariétal Moyen* Can Be Detected in the Depth Profiles of the Central Sulcus

From 1109 subjects (503 male, 606 female; age in years: 28.80 ± 3.70), a total of 2165 CS depth profiles (1080 left hemispheric datasets; 1085 right hemispheric datasets) were surveyed to describe the general pattern of the CS depth trajectory (Table 1), and to identify the PPfpm.

**FIGURE 2:**
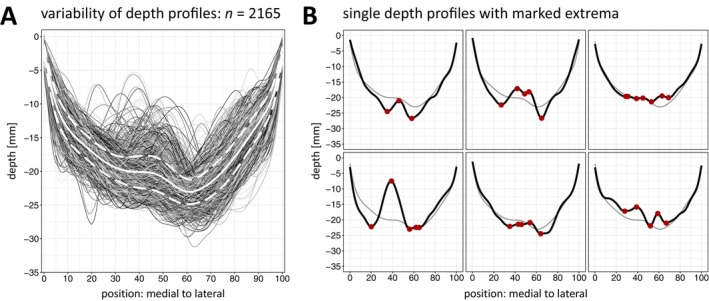
Depth profiles of the central sulcus. Depth [mm] from surface to fundus is given at 101 discrete positions along the CS from medial (*x* = 0; at the interhemispheric cleft) to lateral (*x* = 100; at the lateral sulcus). (A) Superimposed depth profiles of 2165 left (*n* = 1080) and right (*n* = 1085) hemispheric datasets with average depth (white line) and standard deviation of all depth values (dashed line) computed at each position. (B) Six individual depth profiles: Upper row—left hemispheric datasets; bottom row—right hemispheric datasets. Red dots: Extrema in individual depth profiles; grey line: Average depth profile.

The average outline across all depth profiles comprises a U‐shaped trajectory of cortical depth (Figure 2A). Starting medially at position 0, a moderate decline toward the deepest point at position 61 of −23.04 ± 1.83 mm depth is continued by a comparably steep incline in the lateral third, ending at position 100. Central in the depth profiles, a profound but demarked elevation starts in the medial decline and ends in a clearly accentuated drop at the deepest point of the average depth profile, thus discontinuing the otherwise U‐shaped depth trajectory. This elevation, the PPfpm, is on average of only limited height. However, a high degree of variation of the PPfpm's extent and position in the CS depth profiles is indicated by the high deviation of depth and positional values of the PPfpm's maximal height (standard deviation: 2.49 mm; positions: 45–47).

Even though the described depth outline with the centrally located PPfpm can be identified across all 2165 depth profiles, case‐by‐case inspection stresses the high variability of depth profiles, impeding the identification of common but distinct features for automated PPfpm detection. Individual depth profiles vary in extent and shape of the PPfpm from relatively small (Figure 2B, upper left) to almost reaching surface levels (Figure 2B, lower left), including single or double elevations, either pronounced (Figure 2B, upper middle) or comparatively indistinct (Figure 2B, lower middle). Depth profiles diverge in overall shape, presenting with a flat trajectory with none or multiple shallow elevations (Figure 2B, upper right) or multiple distinct elevations (Figure 2B, lower right).

### 3.2 Survey of All Depth Profiles' Extrema Indicates a High Stability of the Global Minimum

**FIGURE 3:**
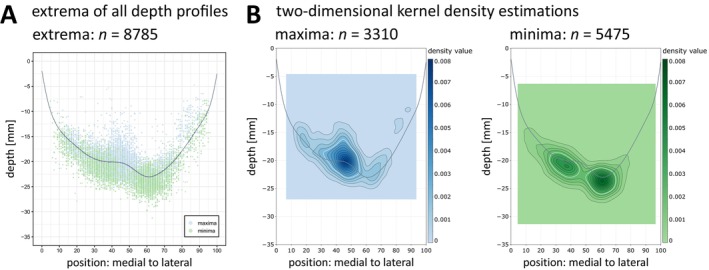
Extrema of all depth profiles (*n* = 2165). (A) Individual maxima (*n* = 3310, blue) and minima (*n* = 5475, green) of 2165 depth profiles. (B) Two‐dimensional kernel density estimation of all maxima (left, blue) and all minima (right, green). Two‐dimensional kernel density estimation is evaluated on a square grid (100 × 100) as defined by the depth values and positions of each the outermost extrema. Contours of density estimation are given at 0.0005 intervals; the white contour line marks the density value 0.0025. Grey line: Average depth profile; blue: Maxima; green: Minima.

For an automated extraction of the PPfpm from the depth profiles, the depth profiles' extrema (*n* = 3310 maxima; *n* = 5475 minima; from *n* = 2165 depth profiles) were identified as stable features within depth profiles, presenting as three prominent clusters along the average depth profile (Figure 3A). A large, spread‐out cluster of maxima is associated with the PPfpm's maximal height. It is adjoined by and partly overlapping with a distinct medial and a distinct lateral cluster of minima.

To deduce the feasibility of these clusters for automated PPfpm detection and to identify the cluster with the most uniform distribution, two‐dimensional kernel density estimations (Figure 3B) of position vs. depth value were performed for all maxima (Figure 3B, left) and for all minima (Figure 3B, right). The density estimation of all maxima reveals a demarked peak incidence distinctly relating to the peak height of the PPfpm in the average depth profile (maximal density value: 0.0072; position: 46; depth: −20 mm). The estimation, however, implies a high dispersal of position and depth values (density value: 0.0025; positional range: 32 to 55; depth range: −16 mm to −23 mm). Contrastingly, the two‐dimensional kernel density estimation of all minima reveals a narrower distribution for both clusters of minima. While the medial cluster is spatially less focused (density value: 0.0025; positional range: 26 to 45; depth range: −19 mm to −23 mm) and comparably attenuated (maximal density value: 0.0046; position: 37; depth: −21 mm), the lateral cluster demonstrates a highly circular density estimation (density value: 0.0025; positional range: 50 to 69; depth range: −21 mm to −26 mm) with a clearly demarked peak density value (maximal density value: 0.0066; position: 61; depth: −24 mm).

**FIGURE 4:**
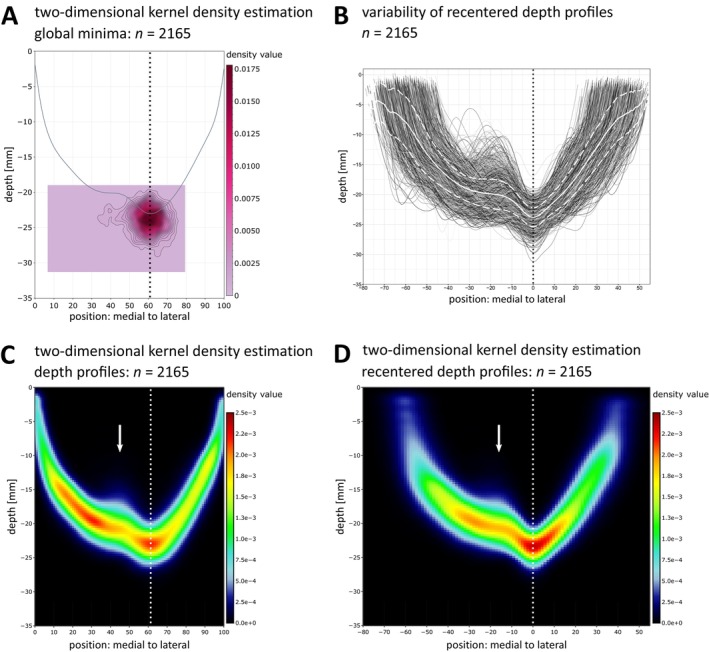
Relation between depth profiles and PPfpm‐II (*n* = 2165). (A) Two‐dimensional kernel density estimation of all global minima in depth profiles. Two‐dimensional kernel density estimation is evaluated on a square grid (100 × 100) as defined by the depth values and positions of the outermost global minima. Contours of density estimation are given at 0.0005 intervals. Grey line: Average depth profile. (B) Superimposed depth profiles after recentering on the respective PPfpm‐II (position 0: PPfpm‐II; negative values: Medial to PPfpm‐II; positive values: Lateral to PPfpm‐II) with average depth (white line) and standard deviation (dashed line) of all depth values computed at each recentered position. PPfpm‐II is thereby defined as the deepest local minimum lateral to position 45. (C) Two‐dimensional kernel density estimation of all depth profiles before and (D) after recentering on PPfpm‐II. Arrow: Peak elevation of PPfpm in two‐dimensional kernel density estimation across all depth profiles. Dotted line: Average position of PPfpm‐II before (A, C: Position: 61) and after (B, D: Position: 0) recentering.

Of all three clusters, the lateral cluster of minima is thus suggested to be of a comparably high invariance in both position and depth value. Further evaluation of this cluster against the average depth profile reveals a close correspondence to the deepest point of the average depth profile, thus indicating the lateral cluster to correspond to the deepest points of individual depth profiles. Two‐dimensional kernel density estimation of the global minima (Figure 4A) confirms a high overlap, revealing a narrow and uniform distribution of global minima with a high peak density value (density value: 0.0178; position: 61; depth: −24 mm). A small divergence from this focalized cluster toward medial positions is caused by 86 global minima at position ≤ 45.

Together, these findings implicate global minima at positions > 45 as highly stable within depth profiles, demarking the PPfpm elevation at its lateral end.

### 3.3 Identification of Stable Features of the *Pli‐de‐Passage Fronto‐Pariétal Moyen*

#### 3.3.1 “PPfpm‐II” in Depth Profiles Represents the Lateral End of the Anatomical *Pli‐de‐Passage Fronto‐Pariétal Moyen*

**FIGURE 5:**
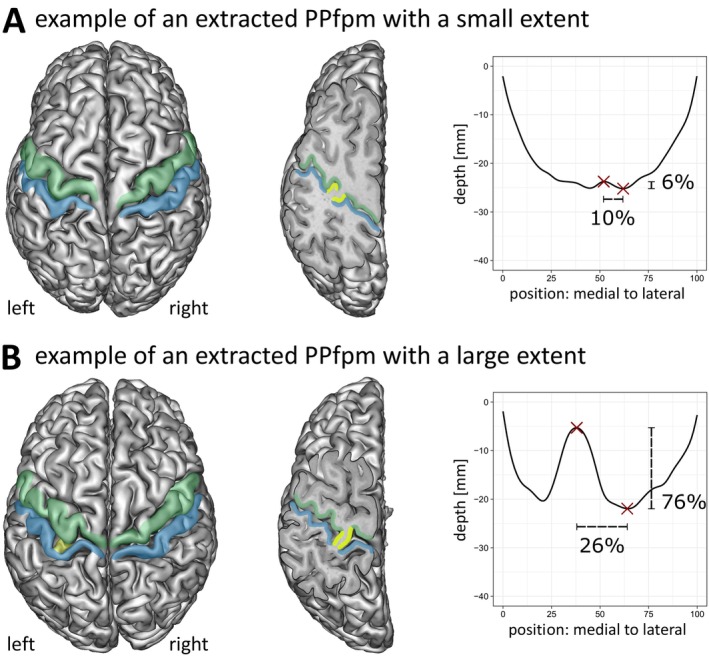
Relation between cortical anatomy and depth profiles. For two exemplary subjects of the HCP‐YA, cortical grey matter reconstructions are shown in relation to depth profiles with extracted PPfpm‐I/PPfpm‐II. Subjects display a PPfpm of (A) small and (B) large extent in the left hemisphere. Left: Cortical grey matter reconstructions with marked precentral gyrus (green), postcentral gyrus (blue) and PPfpm (yellow). Note that the PPfpm is not visible in cortical grey matter reconstructions when its extent is small, but is apparent when its extent is large. Middle: Left hemispheric reconstruction with an oblique slicing plane from superomedial to inferolateral in parallel to the CS fundus reveals the PPfpm (yellow) in both subjects. Note that the depth of the oblique slicing plane is different due to the difference in PPfpm extent. Right: Depth profiles of the left hemisphere with PPfpm‐I/‐II (red crosses) and with computed PPfpm peak‐to‐lateral width and relative PPfpm height indicated in percent.

The lateral end of the PPfpm is marked as feature “PPfpm‐II” and is defined as the global minimum at position > 45 in depth profiles. PPfpm‐II is usually located at the deepest point of the CS depth profile. Anatomically, PPfpm‐II thus corresponds to the apparently highly stable lateral end of the anatomical PPfpm, usually coinciding with the deepest point of the CS (Figure 5, right).

Given the observed stability of PPfpm‐II, the identification of additional PPfpm‐related features in depth profiles was anchored on the position of PPfpm‐II. The benefit of PPfpm‐II as anchor is visualized when individual depth profiles are centered on the PPfpm‐II position (Figure 4B). In these recentered profiles, the PPfpm‐elevation is more clearly demarked. While positional and height variability close to the PPfpm peak height (standard deviation: 2.46 mm; positions: −18 to −19) is retained, the entire PPfpm elevation appears more articulate in comparison to original depth profiles (Figure 2), and—per definition—the average deepest point/lateral end at −23.80 ± 1.60 mm is more accentuated. Two‐dimensional kernel density estimations of all depth profiles before (Figure 4C) and after (Figure 4D) recentering underline this observation. The density estimation of original depth profiles validates the previously described U‐shaped depth trajectory with its focalized deepest point (dotted line) and demonstrates the variability of the PPfpm elevation by a decreased density (arrow). However, after recentering, the PPfpm elevation (arrow)—not the underlying U‐shaped depth trajectory—is more homogenous and overall denser.

Thus, extraction of additional PPfpm features is suggested to be more consistent and reliable when defined in relation to PPfpm‐II.

#### 3.3.2 “PPfpm‐I” in Depth Profiles Represents the Peak Height of the Anatomical *Pli‐de‐Passage Fronto‐Pariétal Moyen*

A second feature, “PPfpm‐I”, identifies the peak height of the PPfpm in depth profiles and is extracted as the local maximum medial to PPfpm‐II. Since the PPfpm occurs in various shapes, PPfpm‐I is defined to consistently identify the peak height directly adjacent to PPfpm‐II. Anatomically, PPfpm‐I identifies the elevation medially closest to the usually deepest point of the entire CS. PPfpm‐I matches the maximal height of the PPfpm in the majority of cases (Figure 5, right).

#### 3.3.3 A Reliable Identification of the Medial End of the *Pli‐de‐Passage Fronto‐Pariétal Moyen* in Depth Profiles Cannot Be Attained

Extraction of a third feature demarking the PPfpm toward its medial end was inquired, since the density estimation of minima (Figure 3C) and the density estimation across all depth profiles (Figure 4C, left) suggested the possibility to define a third feature from depth profiles. However, case‐by‐case inspection found the medial cluster of minima (Figure 3C) to include, but not be limited to, features locating to or demarking the medial end of the PPfpm. Figure 2B provides two examples for this (upper right, lower middle). An accurate match of a third feature to intended anatomical features, that is, the medial end of the PPfpm, could thus not be guaranteed for an adequate number of depth profiles in these large data.

#### 3.3.4 Quality Control of the Identified Features PPfpm‐I and PPfpm‐II

The features PPfpm‐I/‐II were identified and automatically extracted from 2165 depth profiles to enable an observer‐independent characterization of the PPfpm.

Of 2165 analyzed datasets, 1983 depth profiles (91.60%; left hemispheric datasets: *n* = 1008; right hemispheric datasets: *n* = 975) exhibit both PPfpm‐I and PPfpm‐II. While PPfpm‐II could be identified in all 2165 depth profiles, PPfpm‐I is absent in 182 depth profiles (left hemisphere: *n* = 72; right hemisphere: *n* = 110) due to an overall too flat depth profile without any maxima, or exclusion of present maxima as lateral—not medial—to PPfpm‐II.

To confirm the matching of both PPfpm‐I and PPfpm‐II with their anatomical equivalent (Figure 5), individual depth profiles and their extracted features were visually validated against CS meshes, white and grey matter reconstructions and structural MRI data. Manual quality control revealed an accurate detection of PPfpm‐I and PPfpm‐II for 2019 datasets (93.30%; left hemispheric datasets: *n* = 1006; right hemispheric datasets: *n* = 1013), and an erroneous matching of at least one feature in 143 datasets (6.61%). For three datasets, a matching could neither be confirmed nor contradicted (0.14%). Of the 2019 correct depth profiles from 1098 subjects, a subset of 1848 depth profiles (91.53%) from 1070 subjects showed both PPfpm‐I and PPfpm‐II extracted correctly (Table 2), enabling a full characterization of the PPfpm.

**TABLE 2:** Hemispheric datasets after additional quality control of extraction method.

Subjects						Hemispheres		
Groups	Brains (*n*)	Age				Datasets (*n*)		
		M ± SD	SEM	Min	Max	Total	Right	Left
Total	1070	28.82 ± 3.69	0.11	22	37	1848	912	936
Male	486	27.93 ± 3.60	0.16	22	37	860	424	436
Female	584	29.56 ± 3.59	0.15	22	36	988	488	500

Abbreviations: M, mean; SD, standard deviation; SEM, standard error of the mean.

### 3.4 Characterizing the *Pli‐de‐Passage Fronto‐Pariétal Moyen* in the Depth Profiles

Three‐way mixed‐effects model ANOVA statistical analyses were performed on a subset of 778 subjects (*n* = 1556 depth profiles) to examine possible influences on position and depth of PPfpm‐I/‐II, and on the derived PPfpm extent (peak‐to‐lateral width, relative height) by hemisphere (left, right), handedness (left‐handed, right‐handed), and sex (male, female). For these analyses, subjects were considered only if both PPfpm‐I and PPfpm‐II were extracted correctly for both hemispheres (Table 3).

**TABLE 3:** Hemispheric datasets for statistical analysis. Both left and right hemispheric datasets exhibit correctly extracted PPfpm‐I and PPfpm‐II.

Subjects						Hemispheres		
Groups	Brains (*n*)	Age				Datasets (*n*)		
		M ± SD	SEM	Min	Max	Total	Right	Left
Total	778	28.85 ± 3.70	0.13	22	37	1556	778	778
Male	374	28.09 ± 3.61	0.19	22	37	748	374	374
Female	404	29.54 ± 3.65	0.18	22	36	808	404	404

Abbreviations: M, mean; SD, standard deviation; SEM, standard error of the mean.

#### 3.4.1 Position and Depth of the Features PPfpm‐I/‐II

**FIGURE 6:**
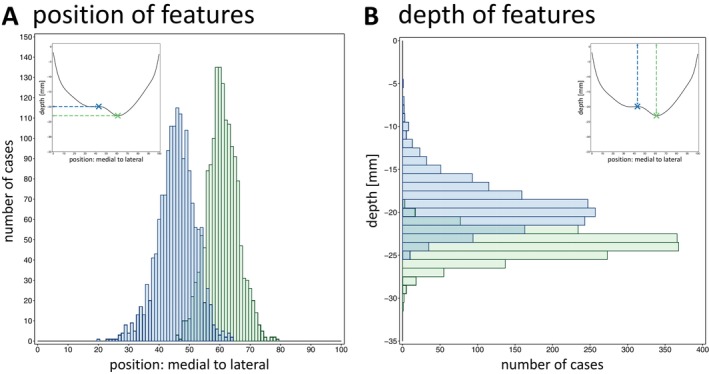
Position and depth values of features PPfpm‐I/‐II. Position (A) and depth [mm] (B) of PPfpm‐I (blue) and PPfpm‐II (green) for 778 subjects (*n* = 1556 depth profiles) where both PPfpm‐I and PPfpm‐II are extracted correctly in both hemispheres. Insets of the average depth profile with marked PPfpm‐I/‐II are shown in the upper corners for comparison.

PPfpm‐I is on average located in the medial half of the CS (position 45.77 ± 6.12) with an average depth of −19.25 ± 2.74 mm (Figure 6, blue). PPfpm‐II is located at the lateral third of the CS (position 60.79 ± 5.12) with an average depth of −23.78 ± 1.60 mm (Figure 6, green). PPfpm‐II closely correlates with the deepest point of the CS at position 59.67 ± 7.32 and with an average depth of −23.82 ± 1.59 mm (Table 4).

**TABLE 4:** Descriptive statistics of depth profiles (*n* = 1556 datasets).

	Position			Depth [mm]		
	PPfpm‐I	PPfpm‐II	Deepest point	PPfpm‐I	PPfpm‐II	Deepest point
M ± SD	45.77 ± 6.12	60.79 ± 5.12	59.67 ± 7.32	−19.25 ± 2.74	−23.78 ± 1.60	−23.82 ± 1.59
SEM	0.16	0.13	0.19	0.07	0.04	0.04
Min	20	46	7	−5.30	−19.04	−19.04
Max	64	79	79	−25.17	−31.24	−31.24

Abbreviations: M, mean; SD, standard deviation; SEM, standard error of the mean.

#### 3.4.2 Position of PPfpm‐I/‐II

**FIGURE 7:**
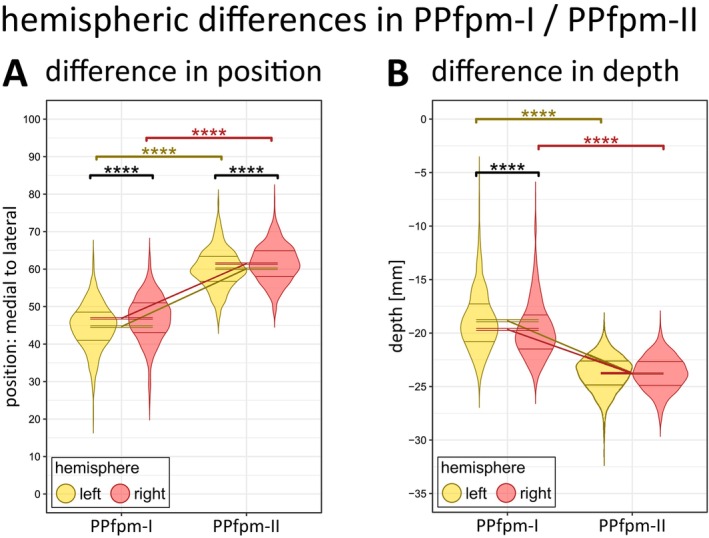
Hemispheric differences (left, right) in features PPfpm‐I/‐II, each in their position (A) and in their depth (B). Distribution of individual values is shown as violin plot (Gaussian kernel) with 0.25 and 0.75 quartile indicated by horizontal lines; M ± SEM are given by hemisphere for PPfpm‐I/‐II. Significance of effects: Post hoc paired *t*‐tests (Bonferroni correction: *p*_crit_ < 0.0125) indicate a significant effect of hemisphere and feature on positional and depth values: *p < 0.0125, **p < 0.0025, ***p < 0.00025, ****p < 0.000025, ns not significant. Yellow: Left hemisphere; red: Right hemisphere.

Analyses on the features' position (Table 5) confirm PPfpm‐I—per definition—significantly more medial than PPfpm‐II. Both PPfpm‐I and PPfpm‐II are more lateral in right hemispheres (Figure 7A) with the effect more apparent on PPfpm‐I (PPfpm‐I: *Δ* = 2.21 positions; PPfpm‐II: *Δ* = 1.38 positions; Bonferroni correction: *p*_crit_ < 0.0125; Table 6). In contrast, PPfpm‐II demonstrates a higher positional stability across hemispheres and subjects as indicated by the smaller effect size and the overall narrower distribution.

**TABLE 5:** Mixed‐effects model ANOVA on position of features PPfpm‐I/‐II (*n* = 1556 datasets).

Effect	$F\left({\mathrm{DF}}_{\mathrm{num}},{\mathrm{DF}}_{\mathrm{den}}\right)$	p	
Feature	$F\left(1,776\right)=10209.96$	<0.0001	****
Hemisphere	$F\left(1,776\right)=66.47$	<0.0001	****
Sex	$F\left(1,776\right)=0.11$	0.75	ns
Feature : hemisphere	$F\left(1,776\right)=1.07$	0.001	**
Feature : sex	$F\left(1,776\right)=3.26\;$	0.07	ns
Hemisphere : sex	$F\left(1,776\right)=0.22\;$	0.64	ns
Feature : hemisphere : sex	$F\left(1,776\right)=0.11$	0.73	ns

*Note:* Significance: *p < 0.05, **p < 0.01, ***p < 0.001, ****p < 0.0001, ns not significant.

Abbreviations: den, denominator; DF, degrees of freedom; num, numerator.

**TABLE 6:** Bonferroni corrected post hoc testing of position of features PPfpm‐I/‐II per hemisphere (*n* = 1556 datasets).

Condition	Comparison	Position				Paired two‐sided *t*‐test			95% confidence interval	
		M	SD	SEM	∆	*t*	p		Lower	Upper
PPfpm‐I	Left	44.67	6.03	0.22	−2.21	−7.86	<0.000025	****	−2.76	−1.66
	Right	46.88	6.02	0.22						
PPfpm‐II	Left	60.10	5.22	0.19	−1.38	−6.15	<0.000025	****	−1.81	−0.94
	Right	61.48	4.93	0.18						
Left	PPfpm‐I	44.67	6.03	0.22	−15.44	−79.97	<0.000025	****	−14.99	−14.21
	PPfpm‐II	60.10	5.22	0.19						
Right	PPfpm‐I	46.88	6.02	0.22	−14.60	−73.81	<0.000025	****	−15.81	−15.06
	PPfpm‐II	61.48	4.93	0.17						

*Note:* Bonferroni adjusted significance: *p < 0.0125, **p < 0.0025, ***p < 0.00025, ****p < 0.000025, ns not significant.

Abbreviations: ∆, estimation of difference of means between comparisons; M, mean; SD, standard deviation; SEM, standard error of the mean.

Handedness of subjects (left‐handed: Oldfield Index < 0; *n* = 67; right‐handed: Oldfield Index > 0, *n* = 708) does not appear to significantly affect the position of PPfpm‐I and PPfpm‐II.

Balanced resampled mixed‐effects model ANOVA (*n* = 10.000 iterations) shows no median main effect of handedness [median “handedness”: $F\left(1,130\right)=0.17,p=0.68$] on the position of PPfpm‐I/‐II. Interaction effects with hemisphere [median “handedness : hemisphere”: $F\left(1,130\right)=1.13,p=0.29$] and feature [median “handedness : feature”: $F\left(1,130\right)=1.27,p=0.26$] are neither significant nor consistent across iterations. A permutation analysis (*n* = 10.000 permutations) confirms the absence of a significant handedness effect (*p*_*emp* *i* *rical*_ = 0.89). A complementary linear mixed‐effects model shows no main effect of handedness (β=0.03,SE=0.24,t=0.14) and no interpretable or robust interactions with hemisphere (β=−0.39,SE=0.15,t=−2.60) or feature (β=0.22,SE=0.15,t=1.46).

#### 3.4.3 Depth of PPfpm‐I/‐II

Analyses of the depth values (Table 7) implicate PPfpm‐II—per extraction method—significantly deeper than PPfpm‐I. Depth values of PPfpm‐II are indicated as stable across hemispheres (Figure 7B), while the depth of PPfpm‐I is significantly deeper in right hemispheres (PPfpm‐I: *Δ* = 0.82 mm; Bonferroni correction *p*_crit_ < 0.0125; Table 8).

**TABLE 7:** Mixed‐effects model ANOVA on depth of features PPfpm‐I/‐II (*n* = 1556 datasets).

Effect	$F\left({\mathrm{DF}}_{\mathrm{num}},{\mathrm{DF}}_{\mathrm{den}}\right)$	p	
Feature	$F\left(1,776\right)=3765.22$	<0.0001	****
Hemisphere	$F\left(1,776\right)=45.06$	<0.0001	****
Sex	$F\left(1,776\right)=81.54$	<0.0001	****
Feature : hemisphere	$F\left(1,776\right)=4.40$	<0.0001	****
Feature : sex	$F\left(1,776\right)=1.00$	0.32	ns
Hemisphere : sex	$F\left(1,776\right)<0.0001\;$	1.00	ns
Feature : hemisphere : sex	$F\left(1,776\right)=1.08$	0.30	ns

*Note:* Significance: *p < 0.05, **p < 0.01, ***p < 0.001, ****p < 0.0001, ns not significant.

Abbreviations: den, denominator; DF, degrees of freedom; num, numerator.

**TABLE 8:** Bonferroni corrected post hoc testing of depth of features PPfpm‐I/‐II per hemisphere (*n* = 1556 datasets).

Condition	Comparison	Depth value [mm]				Paired two‐sided *t*‐test			95% confidence interval	
		M	SD	SEM	∆	*t*	p		Lower	Upper
PPfpm‐I	Left	−18.84	2.80	0.10	0.82	7.43	<0.000025	****	0.60	1.03
	Right	−19.66	2.62	0.09						
PPfpm‐II	Left	−23.78	1.64	0.06	0.00	−0.04	0.97	ns	−0.1	0.1
	Right	−23.78	1.57	0.06						
Left	PPfpm‐I	−18.84	2.80	0.10	4.94	49.50	<0.000025	****	4.74	5.14
	PPfpm‐II	−23.78	1.64	0.06						
Right	PPfpm‐I	−19.66	2.62	0.09	4.12	45.16	<0.000025	****	3.94	4.30
	PPfpm‐II	−23.78	1.57	0.06						

*Note:* Bonferroni adjusted significance: *p < 0.0125, **p < 0.0025, ***p < 0.00025, ****p < 0.000025, ns not significant.

Abbreviations: ∆, estimation of difference of means between comparisons; M, mean; SD, standard deviation; SEM, standard error of the mean.

Taken together with the statistical analysis of positions of PPfpm‐I/PPfpm‐II, right hemispheres are implicated to exhibit flatter depth profiles with an—on average—deeper and more lateral peak height.

Depth of PPfpm‐I and PPfpm‐II is not shown to be robustly affected by a subject's handedness.

Balanced resampled mixed‐effects model ANOVA (*n* = 10.000 iterations) yields no median main effect of handedness [median “handedness”: $F\left(1,130\right)=3.72,p=0.56$]. Median interaction effects with hemisphere [median “handedness : hemisphere”: $F\left(1,130\right)=0.72,p=0.40$] and feature [median “handedness : feature”: $F\left(1,130\right)=1.27,p=0.26$] are not significant. While permutation analysis (*n* = 10.000 permutations) indicates a weak effect on depth of PPfpm‐I/‐II (*p*_*emp* *i* *r* *i* *cal*_ = 0.02), an interaction with hemisphere was not confirmed (*p*_*empir* *ical*_ = 0.06). A complementary linear mixed‐effects model shows no meaningful main effect of handedness given this large sample size (β=−0.23,SE=0.10,t=−2.29), and no significant interactions with hemisphere (β=0.10,SE=0.06,t=1.80) or feature (β=−0.10,SE=0.06,t=−1.72).

Taken together, subjects' handedness does not appear to affect the position or depth of PPfpm‐I or PPfpm‐II. It therefore seems unlikely that handedness exerts a biologically meaningful influence on the general PPfpm morphology.

#### 3.4.4 Extent of the *Pli‐de‐Passage Fronto‐Pariétal Moyen* in Depth Profiles

**FIGURE 8:**
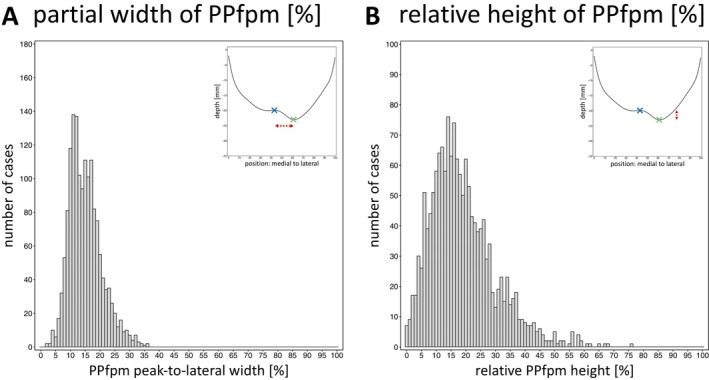
Extent of the PPfpm. Peak‐to‐lateral width (A) and relative height (B) of the PPfpm are given in relation to total CS length and depth of PPfpm‐II. Upper right corner shows the average depth profile for comparison with marked PPfpm‐I (blue) and PPfpm‐II (green), and the observed dimension (red arrow‐line).

The extent of the PPfpm, that is, the peak‐to‐lateral width and height, is derived from the features PPfpm‐I/‐II (Figure 5). The PPfpm peak‐to‐lateral width as the positional difference of PPfpm‐II and PPfpm‐I is given as a relative width of total CS length (15.02% ± 5.47%; Figure 8A). As such, PPfpm peak‐to‐lateral width is defined between the PPfpm's peak height (PPfpm‐I) and lateral end (PPfpm‐II), and not extended to the more intuitive—although unreliable—medial end of the PPfpm. The PPfpm height is the absolute difference in depth between PPfpm‐II and PPfpm‐I (4.53 ± 2.70 mm; Figure 8B). Single case normalization of the absolute PPfpm height to depth of PPfpm‐II gives a relative PPfpm height of 18.94% ± 11.07% (Table 9).

**TABLE 9:** Descriptive statistics of PPfpm extent (*n* = 1556 datasets).

	Peak‐to‐lateral width [%]	Absolute height [mm]	Relative height [%]
M ± SD	15.02 ± 5.47	4.53 ± 2.70	18.94 ± 11.07
SEM	0.14	0.07	0.28
Min	2.00	0.02	0.06
Max	36.00	16.64	75.85

Abbreviations: M, mean; SD, standard deviation; SEM, standard error of the mean.

#### 3.4.5 Peak‐To‐Lateral Width and Relative Height of the *Pli‐de‐Passage Fronto‐Pariétal Moyen* Correlate Strongly

To examine how partial PPfpm width and PPfpm height relate, an exploratory correlational analysis was conducted to assess possible interdependencies between the PPfpm peak‐to‐lateral width and the relative PPfpm height.

**FIGURE 9:**
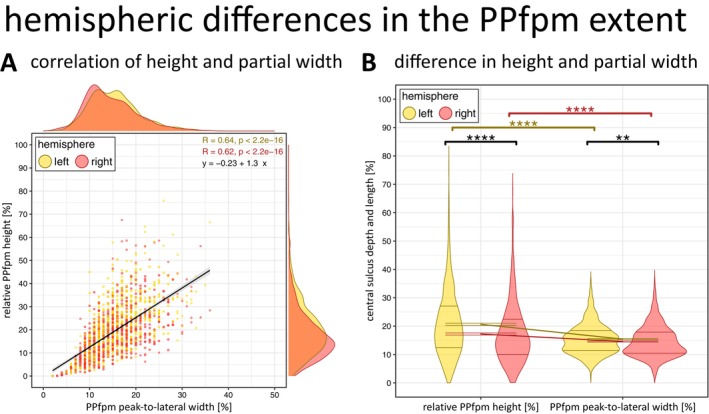
Hemispheric differences (left, right) in the PPfpm extent. (A) Correlation of relative PPfpm height [%] and PPfpm peak‐to‐lateral width [%] for 778 subjects (*n* = 1556 datasets). Note that the peak‐to‐lateral width is defined as positional difference between PPfpm‐II and PPfpm‐I, and thus equals a width from the anatomical PPfpm's lateral end to its peak height. Black line: Regression (linear model) across 1556 datasets with shaded background indicating 95% confidence interval. Pearson's correlation coefficient per hemisphere: *R* = 0.64 (left hemispheric datasets; yellow) and *R* = 0.62 (right hemispheric datasets; red), *p* < 0.0001 indicates a strong correlation. Outer margins: Density (Gaussian kernel) of relative PPfpm height [%] (right), and PPfpm peak‐to‐lateral width [%] (upper) per hemisphere. (B) Hemispheric differences (left, right) in the relative PPfpm height [%] and PPfpm peak‐to‐lateral width [%]. Distribution of individual values is shown as a violin plot (Gaussian kernel) with 0.25 and 0.75 quartile indicated by horizontal lines; M ± SEM are given by hemisphere for dimensions of the PPfpm. Post hoc paired *t*‐test (Bonferroni correction: *p*_crit_ < 0.0125) indicates a significant effect of hemisphere on dimension of PPfpm extent: *p < 0.0125, **p < 0.0025, ***p < 0.00025, ****p < 0.000025, ns not significant. Yellow: Left hemisphere; red: Right hemisphere.

Pearson's correlation coefficient is strong at $R=0.63,p\;\left(\mathrm{two}-\text{tailed}\right)<0.0001$ across all datasets, indicating that higher PPfpms tend to exhibit a greater distance between their peak height (PPfpm‐I) and lateral end (PPfpm‐II). Given the high positional and depth stability of the PPfpm's lateral end, this suggests that the PPfpm's peak height shifts both vertically to higher positions and horizontally toward medial positions as the PPfpm's height increases. Separate analyses by hemisphere (Figure 9A) reveal no relevant hemispheric difference in correlation strength, suggesting that the effect is universal across hemispheres.

Importantly, the observed correlation pertains solely to the PPfpm peak‐to‐lateral width. Since the medial end of the PPfpm cannot be reliably extracted, the full medial to lateral width of the PPfpm and its relationship with the relative PPfpm height cannot be evaluated. Thus, higher PPfpms do not necessarily imply wider PPfpms overall, but only a greater distance between the PPfpm's peak height and its lateral end.

#### 3.4.6 Left Hemispheres Exhibit a Larger *Pli‐de‐Passage Fronto‐Pariétal Moyen*

When quantified relative to CS depth and length, the PPfpm is, on average, higher than its peak‐to‐lateral width in both hemispheres (Table 10; Figure 9B). Compared to right hemispheres, left hemispheres exhibit an overall larger PPfpm, reflected by a greater relative height and a modestly greater peak‐to‐lateral width (relative height: *Δ* = 3.41%; peak‐to‐lateral width: *Δ* = 0.84%; Bonferroni correction *p*_crit_ < 0.0125; Table 11).

**TABLE 10:** Mixed‐effects model ANOVA on extent (peak‐to‐lateral width/relative height) of the PPfpm (*n* = 1556 datasets).

Effect	$F\left({\mathrm{DF}}_{\mathrm{num}},{\mathrm{DF}}_{\mathrm{den}}\right)$	p	
Dimension	$F\left(1,776\right)=268.14$	<0.0001	****
Hemisphere	$F\left(1,776\right)=38.70$	<0.0001	****
Sex	$F\left(1,776\right)=5.25$	0.02	*
Dimension : hemisphere	$F\left(1,776\right)=42.33$	<0.0001	****
Dimension : sex	$F\left(1,776\right)=3.02$	0.08	ns
Hemisphere : sex	$F\left(1,776\right)=0.81$	0.37	ns
Dimension : hemisphere : sex	$F\left(1,776\right)=1.25$	0.26	ns

*Note:* Significance: *p < 0.05, **p < 0.01, ***p < 0.001, ****p < 0.0001, ns not significant.

Abbreviations: den, denominator; DF, degrees of freedom; num, numerator.

**TABLE 11:** Bonferroni corrected post hoc testing of PPfpm extent (peak‐to‐lateral width/relative height) per hemisphere (*n* = 1556 datasets).

Condition	Comparison	Position				Paired two‐sided *t*‐test			95% confidence interval	
		M	SD	SEM	∆	*t*	p		Lower	Upper
Width [%]	Left	15.44	5.38	0.19	0.84	3.29	0.001	**	0.34	1.33
	Right	14.60	5.52	0.20						
Height [%]	Left	20.65	11.41	0.41	3.41	6.92	<0.000025	****	2.45	4.38
	Right	17.24	10.45	0.37						
Left	Width [%]	15.44	5.38	0.19	5.21	16.16	<0.000025	****	4.58	5.85
	Height [%]	20.65	11.41	0.41						
Right	Width [%]	14.60	5.52	0.20	2.64	8.92	<0.000025	****	2.06	3.22
	Height [%]	17.24	10.45	0.37						

*Note:* Bonferroni adjusted significance: *p < 0.0125, **p < 0.0025, ***p < 0.00025, ****p < 0.000025, ns not significant.

Abbreviations: ∆, estimation of difference of means between comparisons; height [%], relative PPfpm height; M, mean; SD, standard deviation; SEM, standard error of the mean; width [%], PPfpm peak‐to‐lateral width.

Together with the influence of hemispheres on PPfpm‐I/‐II, right hemispheres are indicated to—on average—exhibit flatter depth profiles correlating with a more lateral and smaller PPfpm with a lower peak height.

#### 3.4.7 Relative Height of the *Pli‐de‐Passage Fronto‐Pariétal Moyen* Differs Between Males and Females due to Deeper Sulci in Males

**FIGURE 10:**
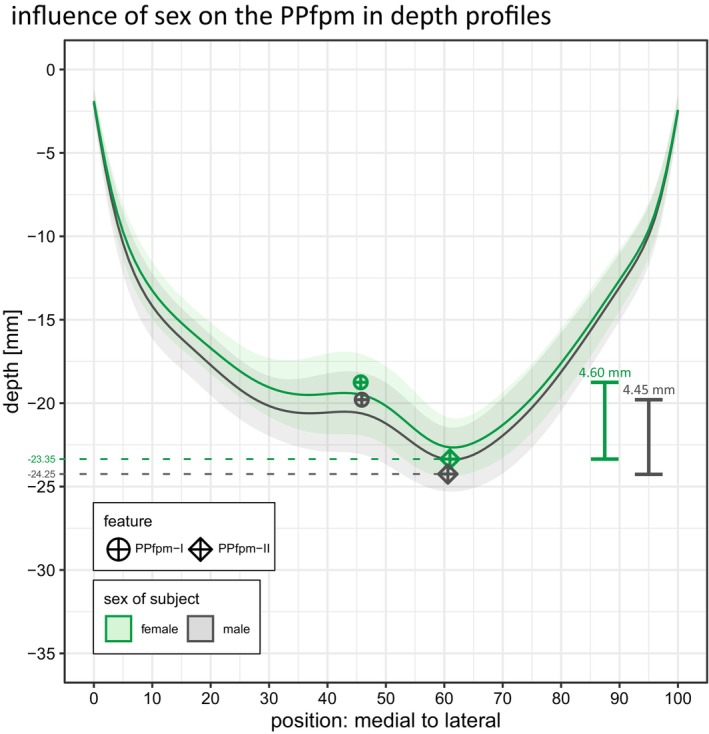
Effect of sex on the PPfpm in depth profiles. Average depth profile of males (grey, *n* = 748 datasets; *n* = 374 subjects) and females (green, *n* = 808 datasets; *n* = 404 subjects) computed per position; shaded area indicates the standard deviation. PPfpm‐I (circle‐cross) and PPfpm‐II (diamond‐cross) are averaged across all male and female datasets.

For both PPfpm‐I and PPfpm‐II, depth but not positional values are significantly different between males (*n* = 374) and females (*n* = 404). Both PPfpm‐I and PPfpm‐II are deeper in males (PPfpm‐I: *Δ* = 1.04 mm; PPfpm‐II: *Δ* = 0.90 mm; Table 12), an effect suggested to originate from overall deeper CS in males (Figure 10A).

**TABLE 12:** Descriptive statistics of PPfpm extent and features PPfpm‐I/‐II by sex (*n* = 1556 datasets).

	Sex	PPfpm extent			Feature			
		Width [%]	Height [mm]	Height [%]	PPfpm‐I		PPfpm‐II	
					Position	Depth [mm]	Position	Depth [mm]
M	Male	14.74	4.45	18.23	45.87	−19.79	60.61	−24.25
	Female	15.28	4.60	19.60	45.69	−18.75	60.96	−23.35
SD	Male	5.46	2.69	10.77	6.21	2.65	5.39	1.60
	Female	5.46	2.71	11.30	6.05	2.73	4.87	1.48
SEM	Male	0.20	0.10	0.39	0.23	0.10	0.20	0.06
	Female	0.19	0.10	0.40	0.21	0.10	0.17	0.05

Abbreviations: height [%], relative PPfpm height; height [mm], absolute PPfpm height; M, mean; SD, standard deviation; SEM, standard error of the mean; width [%], PPfpm peak‐to‐lateral width.

A statistical effect of sex on the extent of the PPfpm is observed (Table 10). After supplementary two‐way mixed‐effects model ANOVA investigating the effect of hemisphere and sex on each the relative height and the peak‐to‐lateral width of the PPfpm, a weak influence of sex is observed for relative PPfpm height only [“sex”: $F\left(1,776\right)=5.03,p=0.03$], but not for PPfpm peak‐to‐lateral width [“sex”: $F\left(1,776\right)=3.26,p=0.07$]. Two‐way mixed‐effects model ANOVA on the influence of hemisphere and sex on the absolute—in contrast to the relative—PPfpm height could not confirm any influence of sex [“sex”: $F\left(1,776\right)=1.00,p=0.32$].

Given the observed depth difference in the depth trajectory between males and females and considering the effect of sex on the depth of PPfpm‐I/PPfpm‐II, the weak effect of sex on relative PPfpm height is implicated to result from the normalization procedure itself. That is, the absolute PPfpm height (Figure 10B; Table 12) is not significantly different between males and females, while the relation to total CS depth of PPfpm‐II is.

## 4 Discussion

In the present study, structural MRI data from 1112 subjects of the HCP‐YA S1200 Release were analyzed to provide a comprehensive macroanatomical characterization of the PPfpm in a large cohort:

A method was devised to extract the PPfpm from CS depth profiles (*n* = 2165), defining two stable key features: the PPfpm's lateral end (PPfpm‐II: global minimum at position > 45) and its peak height (PPfpm‐I: medially adjacent local maximum to PPfpm‐II). The PPfpm was identified as a near universal cerebral fold at the CS fundus at mid‐height: In depth profiles, both PPfpm‐I and PPfpm‐II are detectable in > 90% of depth profiles (*n* = 1983); in structural MRI, the PPfpm was clearly detectable in all but three cases. Analyses confirmed the commonly small height of the PPfpm < 5 mm; its height and peak‐to‐lateral width correlate positively. The PPfpm does, however, exhibit some variability in its shape and extent. Notably, the lateral end of the PPfpm coincides with the deepest point of the CS in > 95% of depth profiles (*n* = 2079). A statistically significant, but minor, difference was found in the PPfpm's position, in its peak height, and in its general extent between left and right hemispheres: On average, left hemispheres exhibit a more medial and higher PPfpm with a greater distance between peak height and lateral end. Handedness of subjects is not shown to affect the PPfpm. Sex of subjects has no effect on the absolute height of the PPfpm.

### 4.1 Quality of Extraction of the *Pli‐de‐Passage Fronto‐Pariétal Moyen* From Central Sulcus Depth Profiles

While the developed method accurately extracted the PPfpm from depth profiles in the majority of cases, there is a non‐negligible number of datasets where extraction of the PPfpm was only partially possible (*n* = 182, 8.41%) and/or incorrect (*n* = 146, 6.74%).

Incomplete extraction was prevalent among very shallow depth profiles with a minuscule PPfpm (< 2 mm height) that did not allow for a precise identification of PPfpm‐I. Additionally, the PPfpm of some depth profiles exhibited a steep incline toward medial, masked by the overall trajectory of the depth profile: Without a distinct peak height, PPfpm‐I could thus not be identified by the implemented extraction method. Erroneous extraction of PPfpm‐I or PPfpm‐II resulted from only minor anatomical inaccuracies, wherein an overall flat depth profile impeded the identification of PPfpm‐II as the deepest point, or where the deepest point of the CS did not match the lateral edge of the PPfpm. In some of these cases, refinement of the applied extraction method with a layered extraction based on a prior clustering of depth profiles according to their main trajectory could have facilitated a more successful extraction. Refinement of the extraction method was, however, not pursued, as incomplete or erroneous extractions were prevalent in < 10% and given that the procedure underwent rigorous, repeated quality control. To ensure maximal intra‐rater consistency across all evaluations, manual quality control was performed by a single experienced rater (AMM); a formal inter‐rater reliability assessment was not conducted. To counteract a possible effect of erroneous/incomplete extraction on the detailed statistical analyses of the PPfpm, all analyses were performed on a subset of 1556 datasets where a correct and complete extraction of the PPfpm was obtained in both hemispheres of a subject. Since characteristics of this subset (e.g., age of subjects, number of male/female subjects) showed no notable deviation from the complete dataset, statistical analyses are expected to be generalizable to the entire data under study.

### 4.2 The Shape of Depth Profiles and of the *Pli‐de‐Passage Fronto‐Pariétal Moyen* Varies

As demonstrated, depth profiles exhibit considerable variation in their overall trajectories. This observation is consistent with the report by McKay et al. (2013), who described three distinct depth profile configurations: unimodal, bimodal, and trimodal.

Specifically, McKay et al. (2013) reported unimodal depth profiles (prevalence: 8%) as lacking a distinct central PPfpm, instead presenting a single deepest point accompanied by a sharp medial incline. This pattern closely resembles configurations observed in the current dataset, in which the PPfpm is embedded within a steep medial incline, occasionally complicating its identification, as discussed in detail above. Bimodal depth profiles are reported by McKay et al. (2013) as most common (70%) and display a clear, singular PPfpm elevation. This configuration corresponds to most cases observed in the present study, as exemplified in Figure 2B (upper left, lower left). Trimodal depth profiles (15%) are described by McKay et al. (2013) to exhibit a double central elevation with multiple candidates for a discernible PPfpm elevation, a pattern found, for example, in Figure 2B (upper right, lower right). Although the categorization of depth profiles was not a primary objective of the current analysis, the observed data do not contradict the classifications proposed by McKay et al. (2013). However, the range of depth profile variability observed here suggests that such configurations may not represent discrete categories but rather points along a continuum reflecting the complex cortical topology of the human brain.

As implicated by McKay et al. (2013), and as shown in this study, the PPfpm itself presents most commonly as a small, singular elevation at central positions within depth profiles. However, a previously unreported portion of depth profiles and their PPfpm transition toward a double PPfpm elevation (< 10%). Here, the PPfpm consists of not one singular, but of two more or less discrete peak heights separated by a local depression, seen, for example, in Figure 2B (middle column). Although these PPfpm shape variations may superficially resemble the “trimodal depth profiles” characterized by two discrete elevations, careful examination of cortical reconstructions and the original structural MRI data reveals that they are, in fact, distinct, with only one of these elevations corresponding to the PPfpm in depth profiles. The origin of this morphological variation remains unclear, and future studies should address this anatomical detail more thoroughly.

Additionally, this variability in PPfpm shape introduces ambiguity in the extraction of stable PPfpm features, particularly regarding the identification of the PPfpm's peak height and its medial end, as multiple candidates may arise for both. To increase the consistency of the extraction of stable PPfpm features, the PPfpm peak height at PPfpm‐I was thus defined as the maximum closest to the stable lateral end of the PPfpm at PPfpm‐II. Extraction of the medial PPfpm end as a third feature was excluded from the analysis to mitigate potential confounding effects introduced by the highly variable double PPfpm and trimodal depth profile shape variants.

### 4.3 The *Pli‐de‐Passage Fronto‐Pariétal Moyen* Is a Near‐Universal Structure of Commonly Small Extent Located at Mid‐Height in the Central Sulcus

The present study confirms that the PPfpm is a near‐universal cerebral fold in the adult human brain. It is clearly detectable in structural MRI and/or derived CS depth profiles in the vast majority of 1112 subjects.

Analyses of the PPfpm's gross morphology highlight its generally small extent in the millimeter range with an average absolute height < 5 mm and relative height < 19%. Notably, a superficial PPfpm reaching the cortical surface and discontinuing the CS was found in 9 of 1112 subjects as reported separately by the authors (Schweizer et al. 2025). Results further demonstrated that the PPfpm height lies on a continuum, ranging from a subtle elevation to rare superficial variants. This aligns with the descriptions of Cunningham (1890), who described that the PPfpm can reach “all gradations between a mere shallowing […] of the [CS] and the presence of a distinct deep annectant gyrus”, and is consistent with a detailed report on the PPfpm height by Heschl (1877), replicated by the authors separately (Schweizer et al. 2025).

The average PPfpm peak‐to‐lateral width equals 15% of total CS length. Due to the methodology employed for the CS parametrization, absolute CS length values cannot be calculated directly, as the measures are taken at discrete, relative positions along the CS' medial to lateral trajectory. Thus, an exact metric measure of the PPfpm's absolute peak‐to‐lateral width is unobtainable. However, with an average CS length of ca. 11.6 cm (White et al. 1997b), the peak‐to‐lateral width of the PPfpm can be extrapolated to approximately 17.4 ± 6.3 mm, suggesting that the PPfpm is—in absolute measures—generally wider than higher. Notably, due to the lack of extraction of the PPfpm's medial end, PPfpm peak‐to‐lateral width was defined as the relative distance from the PPfpm's peak height to its lateral end, and not as the more intuitive distance between the PPfpm's medial and lateral end. As such, this metric captures only the partial, not the full width of the PPfpm. This methodological constraint should be considered when interpreting the findings.

Results further corroborate the consistent and stable positional location of the PPfpm within the CS. Importantly, the positional stability of the PPfpm refers to its relative position within the normalized depth profiles of the CS and does not imply an equally stable location in stereotactic space, as the CS itself differs substantially across individuals and between hemispheres (White et al. 1997a). Notably, despite the PPfpm's variability in extent and peak height, its lateral end exhibits a remarkable positional consistency within the CS across this large cohort.

On average, the PPfpm in depth profiles is consistently located at mid‐height within the CS: the PPfpm's peak height at PPfpm‐I lies at position 46; the PPfpm's lateral end at PPfpm‐II at position 61. At first glance, this mid‐height placement of the PPfpm appears to contrast with prior accounts that place the PPfpm at the superomedial third of the CS (Cunningham 1890; Eberstaller 1890; Skandalakis et al. 2025). However, this discrepancy may be attributable to methodological differences in defining and measuring the CS length. First, in the present study, depth measurements were taken at equidistant positions along the medial to lateral trajectory of the CS, thus compensating for individual variations in CS length and curvature. This level of standardization was likely difficult to achieve in earlier anatomical investigations, particularly those from the 19th century. Second, the intrinsic variability of the CS curvature across individuals complicates visual estimations and may contribute to inconsistencies in previous reports on the PPfpm's localization. Third, the medial and lateral extremities of the CS exhibit substantial anatomical variability, further impeding attempts to localize the PPfpm relative within the CS. In particular, the inferolateral end of the CS—near the lateral sulcus—has been reported to vary considerably between individuals (Eberstaller 1890; Eichert et al. 2021), either being separated from the lateral sulcus by a large subcentral gyrus, or merging superficially with it due to confluence with the anterior subcentral sulcus, historically referred to as the “inferior transverse sulcus of the central sulcus” (“*Untere Querfurche der Centralfurche*”) (Cunningham 1890; Eberstaller 1890; Ecker 1869; Turner 1866). Taken together, while the present findings do not precisely replicate earlier descriptions of the PPfpm's position within the CS, they are reconcilable with them when methodological and anatomical differences are considered.

### 4.4 The *Pli‐de‐Passage Fronto‐Pariétal Moyen* in the Context of the Sulcal Pits of the Central Sulcus

In the present study, extraction of the PPfpm from depth profiles is based on the identification of extrema—specifically a local maximum corresponding to the PPfpm's peak height and local/global minima corresponding to its medial and lateral end. Two prominent clusters of minima are thereby found to flank the average PPfpm in depth profiles at its medial and lateral ends (Figure 3). Although not an a priori goal of this study, these observations closely align with previous reports on the sulcal pits of the CS, prompting further consideration of the relationship between the PPfpm and sulcal pit anatomy.

Sulcal pits are defined as the locally deepest points of the cerebral cortex, located along the sulcal bottom lines within the sulcal basins of each cortical sulcus (Lohmann et al. 2008). They are proposed to be the first locations of cortical folding in the fetal brain, exhibit minimal change throughout cortical development (Lohmann et al. 2008; Régis et al. 2005), and are consistently described as stable anatomical landmarks in the human cerebral cortex. While the number of sulcal pits may vary across individuals and hemispheres, those within primary fissures such as the CS are reported to be stable in both number and position (Hostalet et al. 2025; Meng et al. 2014, 2018).

In the CS, two or three sulcal pits are typically observed in individuals, with the two‐pit configuration being more common (Im et al. 2010; Meng et al. 2014, 2018). These two sulcal pits are usually located in the upper and middle third of the CS (Im et al. 2010; Meng et al. 2018), corresponding to the location of the PPfpm. Their position also aligns with anatomical descriptions by Cunningham (1890), confirmed by Régis et al. (2005) and Cachia et al. (2003), who describe the CS to arise from two primitive folds, or “sulcal roots”. It has therefore been proposed that these two most common CS sulcal pits correspond to the sulcal roots of the CS (Im et al. 2010; Meng et al. 2014). Considering that the PPfpm is described as the “eminence” between the CS sulcal roots, a close association between the PPfpm and the sulcal pits of the CS is both plausible and consistent with prior reports (Im et al. 2010; McKay et al. 2013). Thus, the medial cluster of minima is assumed to correspond to the first, upper sulcal pit, and the lateral cluster of minima is thought to correspond to the second, middle sulcal pit.

In the present data, the lateral cluster of minima—from which PPfpm‐II was derived—was notably more stable in both position and depth, and coincides with the deepest point of the CS. This finding is consistent with observations from Im et al. (2010), who reported a higher density of the corresponding sulcal pit. Notably, Cunningham (1890) described the lower sulcal root of the CS as forming earlier than the upper one, supporting the broader notion that sulcal pits are more stable the earlier they develop. This may further explain why the middle sulcal pit—and thus the lateral cluster of minima—shows greater stability, whereas the medial cluster of minima was not sufficiently consistent to serve as a reliable reference for the medial end of the PPfpm in depth profiles.

Taken together, the present study reinforces the close anatomical association between the PPfpm and the CS sulcal pits. Notably, for other areas in the human brain, sulcal pits have further been closely linked to functional specialization (e.g., Auzias et al. 2015; Im et al. 2010; Lohmann et al. 2008; Natu et al. 2021; Régis et al. 2005). Given additional evidence on the association of the PPfpm with the sensorimotor hand/digit area (Alkadhi and Kollias 2004; Boling and Olivier 2004; Gordon et al. 2023; Jensen et al. 2023; Skandalakis et al. 2025), the need for further investigation on the structure–function relationship between the PPfpm, the CS sulcal pits, and functional regions in S1 and M1 is underscored.

### 4.5 The *Pli‐de‐Passage Fronto‐Pariétal Moyen* Displays Stable Features Across Hemispheres, Handedness and Sex

As outlined above, the PPfpm is a common and usually small structure in the human brain that exhibits some variation in extent and shape. While these variations are generally minor on a gross anatomical scale, the influence of biological factors, that is, the hemispheric location of the PPfpm, and the handedness and sex of the subject, were analyzed:

Regarding the position of the PPfpm, the PPfpm's peak height (PPfpm‐I) and lateral end (PPfpm‐II) are found more lateral in right hemispheres. While significant in these large data, the effect is minor with PPfpm‐I more lateral by < 3 positions, and PPfpm‐II more lateral by < 2 positions. Given reports on the different length of left and right hemispheric CS with left hemispheres indicated to exhibit longer CS (Mashouf et al. 2017; White et al. 1997b), this might reflect the different CS morphology of left versus right hemispheres rather than of the PPfpm itself. However, in the absence of the individual absolute length of the CS for these data, this hypothesis remains speculative. While statistically significant and highlighting intrinsic differences between left and right hemispheric CS, the observed effect lies, nevertheless, within millimeter range and thus underlines the PPfpm's stability within the CS on a gross anatomical scale. Regarding depth values of both the PPfpm's peak height and lateral end, the PPfpm's peak height is found generally deeper in right hemispheres. While the depth of the PPfpm's lateral end (PPfpm‐II) remains consistent across all data and is unaffected by hemispheric location, the PPfpm's peak height (PPfpm‐I) is found to be significantly deeper in right hemispheres, although the difference is minimal at 0.82 mm on average. Together, this suggests that—on average—right hemispheres tend to exhibit a slightly smaller PPfpm that—given the correlation of PPfpm height with PPfpm peak‐to‐lateral width—is narrower between its peak height and lateral end, and generally lies more lateral. Nevertheless, at the gross anatomical level, this effect is estimated to be < 1 mm and thus highlights the overall stable morphology of the PPfpm.

Subjects' handedness is not shown to influence the position or depth of the PPfpm's peak height (PPfpm‐I) or lateral end (PPfpm‐II), suggesting that the PPfpm's morphology is largely independent of handedness‐related lateralization. Although the pronounced imbalance between left‐ and right‐handed subjects in the HCP‐YA sample could, in principle, affect statistical sensitivity, the multilevel analytical approach applied here—combining balanced resampling mixed‐effects model ANOVA, permutation analysis, and linear mixed‐effects modeling—did not reveal a consistent handedness‐related pattern. Nonetheless, the extracted PPfpm features capture only two points of the PPfpm, that is, its peak height and lateral end. As such, subtle morphological differences may have remained undetected. Given the PPfpm's proximity to the hand knob of the precentral gyrus, for which handedness‐related asymmetries have been reported (Coulon et al. 2015; Sun et al. 2012), future investigations should examine their spatial relationship in greater detail to assess potential, subtle handedness‐related effects.

Generally, the PPfpm was found to be unaffected in its absolute height by the subjects' sex with an average absolute height difference of 0.15 mm between males and females. In contrast, the relative PPfpm height was found significantly smaller in male brains, though the effect is minor (< 1.5%). This difference is likely attributable to the CS being, on average, ca. 1 mm deeper at PPfpm‐I/‐II positions in males. Due to the absence of precise, modern data on the PPfpm during prenatal development and the lack of longitudinal information across the lifespan, it remains unclear when the PPfpm reaches its mature adult form, and whether this timing differs between sexes. While the consistent absolute height of the PPfpm across subjects is a noteworthy finding, its underlying origin remains uncertain.

In summary, while statistically significant, differences observed in the PPfpm's extent and in the position and depth of PPfpm‐I/‐II were all minor in magnitude. All reported effect sizes fall within or only slightly exceed the spatial resolution of the original MRI data, suggesting that these variations may, at least in part, reflect limitations in measurement precision. These findings thus reinforce that the PPfpm is a common, generally small, and structurally consistent cerebral fold within the human CS, and they highlight the remarkable macroanatomical stability of the PPfpm.

### 4.6 Limitations

A key limitation of the present work is that it does not extend beyond the macroanatomical characterization of the PPfpm, lacking (A) cytoarchitectonic data specific to the PPfpm, and (B) a direct assessment of its association with the functional sensorimotor hand/digit area.

Given its location at the CS fundus, the PPfpm is likely situated near BA4 (i.e., M1 in the precentral gyrus) and BA3 (i.e., S1 in the postcentral gyrus). Cytoarchitectonic investigations of the CS by White et al. (1997a) support this interpretation, showing that at the height of the PPfpm (i.e., “a region of increased complexity”), BA4 extends along the posterior wall of the precentral gyrus and toward its base, where it transitions to BA3, which extends along the anterior wall of the postcentral gyrus. Although this BA4/BA3 border is described as comparatively consistent along the CS (White et al. 1997a), its precise location nonetheless varies substantially between individuals and hemispheres and may deviate by up to 1 cm relative to the CS fundus (Rademacher et al. 2001; White et al. 1997a). Recent work has similarly linked the *pli‐de‐passages*, including the PPfpm, to the BA4/BA3 transition zone (Skandalakis et al. 2025). Nevertheless, the substantial interindividual and intraindividual variability of the BA4/BA3 border (Rademacher et al. 2001; White et al. 1997a), and the scarcity of cytoarchitectonic studies examining the PPfpm directly, leave its precise cytoarchitectonic composition unresolved. Thus, although the present study found the PPfpm to be positionally stable within the CS, the PPfpm cannot be assumed to align equally stably or reliably with cytoarchitectonically distinct areas.

Likewise, although the PPfpm has been proposed as a potential anatomical landmark for the functional sensorimotor hand/digit area, the present study includes no functional neuroimaging or electrophysiological data to test this possibility. Future work will address this limitation by integrating functional data with the anatomical framework established here.

As the present study is conducted on a large dataset with > 1000 subjects from a diverse ethnic background, it is reasonable to assume that the presented results reflect the general characteristics of the PPfpm in the general population. Given that data were obtained exclusively from healthy subjects, the PPfpm's structural characteristics might, however, differ in a patient population where organic brain disorders might affect the general brain morphology. Additionally, with the dataset restricted to young adults, it remains unknown how the PPfpm might be affected by age‐related changes of cortical morphology. With ample evidence of a general decrease of cortical volume and surface in healthy aging adults, including the pre‐ and postcentral gyri (Fleischman et al. 2014; Zheng et al. 2019), it is reasonable to assume that the PPfpm itself might be affected by cortical thinning and decrease in total volume with increasing age. A translation of the PPfpm's detailed characteristic to a clinical or aging population must thus be done cautiously. While the inclusion of additional structural MRI data, for example, of the Chinese Human Connectome Project, the UK Biobank, or the Adolescent Brain Cognitive Development study, could have broadened the generalizability of the findings, the decision to focus exclusively on the HCP‐YA data was made to ensure a high and consistent data quality and comparable preprocessing standards. Incorporating heterogeneous data would have introduced variability, potentially obscuring subtle structural features of the PPfpm. Further, a key strength of the present study is the rigorous quality control applied to each dataset individually, a standard that would be difficult to maintain in larger, more diverse populations.

A methodological limitation of the present study is that the detailed characterization of the PPfpm was conducted on computed CS depth profiles derived from structural MRI data and is based neither directly on structural MRI in native space nor on true anatomical observations.

Specifically, the analytical framework operates on depth profiles derived from relational graph structures of the CS and its rendered mesh, rather than on the original structural MRI data in native space. As such, PPfpm features extracted from depth profiles cannot be directly mapped to native space or projected to standardized stereotactic space (e.g., Montreal Neurological Institute space). While this constraint does not affect the validity of the presented morphological findings, future work should aim to enable subject‐wise extraction of PPfpm features directly from structural MRI data, rather than relying on normalized CS depth profiles.

Additionally, although the present approach enables the precise and observer‐independent characterization of the PPfpm in a large cohort (Cykowski et al. 2008), the resulting morphological and statistical analyses are derived from MRI‐based abstractions rather than direct anatomical observations. Minor differences between the characterization of the PPfpm reported here and those in historic (Heschl 1877) and contemporary (Skandalakis et al. 2025) anatomical studies are thus likely. While a case‐by‐case validation of the PPfpm's morphology in depth profiles versus true anatomical study would be ideal, it is—at present—unavailable.

Nevertheless, the key findings of this study, that is, the universality of the PPfpm, its small extent, its distinct shape as a cerebral fold, and its high positional stability within the CS across hemispheres, handedness, and sex, are unlikely to be contradicted by a direct anatomical comparison. In fact, the general description of the PPfpm as a common structure with small but variable extent has recently been replicated across time in an independent sample (Schweizer et al. 2025).

### 4.7 Anatomical Location and Identification of the *Pli‐de‐Passage Fronto‐Pariétal Moyen* in Structural Imaging Data

To ease the identification of the PPfpm on structural imaging data, this last section summarizes and describes how to best identify the PPfpm on structural MRI data in two exemplary subjects of the HCP‐YA (for comparison of depth profile data see the same two subjects in Figure 5).

**FIGURE 11:**
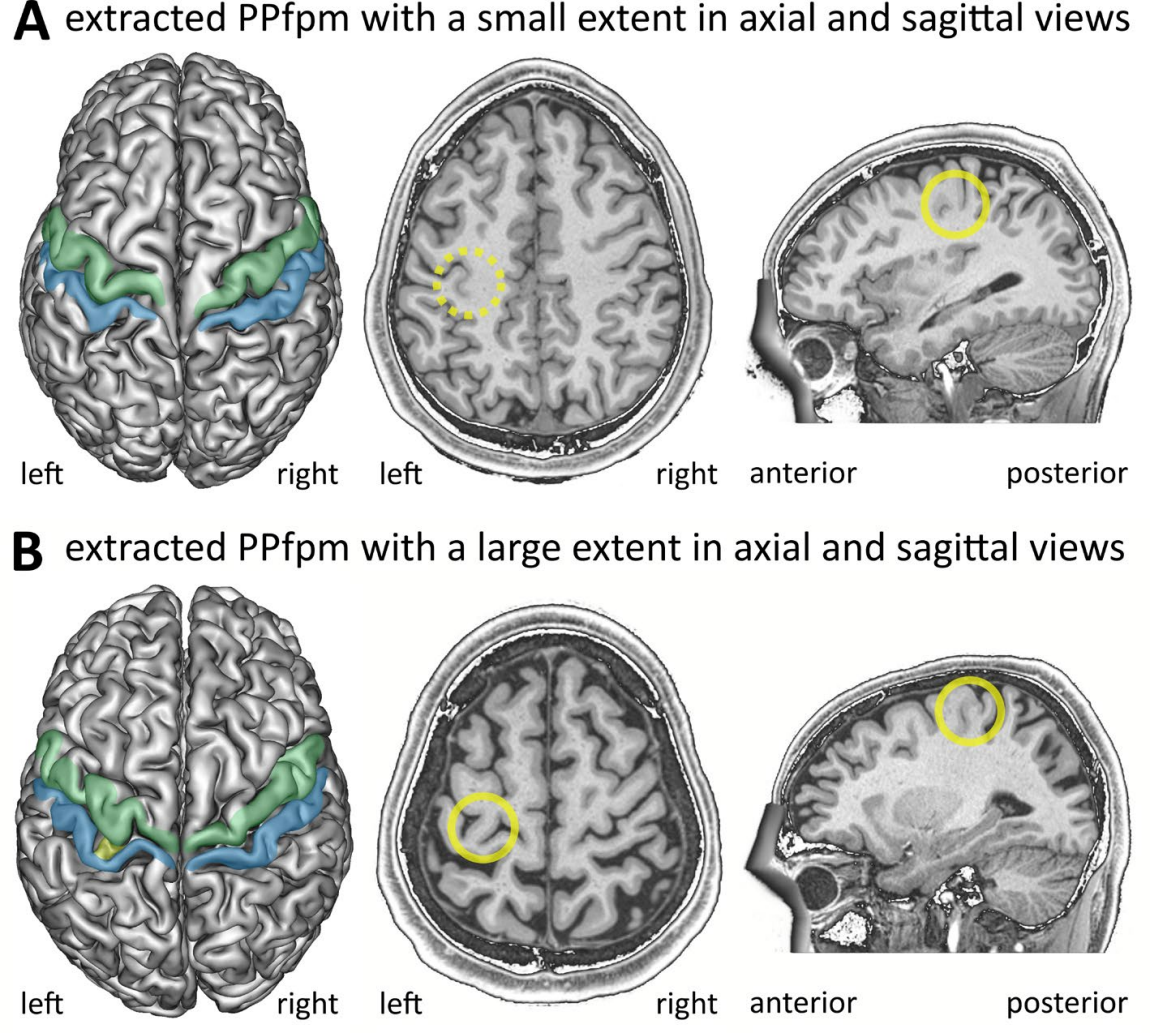
The PPfpm as seen on the cortical surface and in standard MRI views in native space. For two exemplary subjects of the HCP‐YA, cortical grey matter reconstructions are shown in relation to the anatomy as seen in standard axial and sagittal MRI. Subjects display a PPfpm of (A) a small and (B) a large extent in the left hemisphere. Left: Cortical grey matter reconstructions with marked precentral gyrus (green), postcentral gyrus (blue) and PPfpm (yellow). Note that the PPfpm is not visible in cortical grey matter reconstructions when its extent is small but is apparent when its extent is large (B). Middle: Axial MRI view with the PPfpm marked with a yellow circle. Note that a PPfpm with a small extent (A) is not clearly demarked in axial views; the dotted circle indicates the position of the PPfpm. Only a PPfpm with a large extent (B) can be visualized as a distinct cerebral fold. Right: Sagittal MRI view with the PPfpm marked in a yellow circle. The PPfpm is generally detectable as a distinct connection between the precentral hook of the hand knob and a counter‐hook of the postcentral gyrus. Note that the PPfpm can be detected in sagittal views even if it is of only a small extent.

First, the PPfpm is generally located at mid‐height in the CS and shows a close spatial relationship with the hand knob. On cortical surface reconstructions (Figure 11, left), it is located close to the middle frontal gyrus, and—depending on the overall trajectory and length of the CS—lies centrally or at the superomedial third of the CS. Generally, the PPfpm can be seen at the cortical surface only if of an unusually large extent (Figure 11B), or in the rare case of a superficial PPfpm (Schweizer et al. 2025). Second, the PPfpm lies obliquely and roughly perpendicular to the main trajectory of the CS (Heschl 1877). With the CS following a main direction from superior to inferior, posterior to anterior, and medial to lateral, the PPfpm traverses from post‐ to precentral gyrus from inferior to superior, posterior to anterior, and lateral to medial. Given this oblique orientation of the PPfpm, only in some cases can it be observed in axial view, that is, when of a large extent (Figure 11B, middle) but not when of a small extent (Figure 11A, middle), or when the CS presents with a less oblique main trajectory from superior to inferior. Third, the PPfpm can be best identified in sagittal view. Here it presents in the vast majority of cases as an unambiguous connection between pre‐ and postcentral gyrus traversing the CS: Thereby, the hook structure (Yousry 1997) of the hand knob connects with a similar counter‐hook on the postcentral side, forming a—in sagittal view—free‐standing connection.

## 5 Conclusion

In conclusion, the present study provides a comprehensive characterization of the *pli‐de‐passage fronto‐pariétal moyen* (PPfpm), a deep cerebral fold hidden in the depth of the central sulcus (CS). Identified as a near‐universal structure of the human brain, the PPfpm consistently exhibits two stable features: its peak height, and its lateral end at the deepest point of the CS. Both features were subject to extensive quality control. By examining a large cohort of 1112 subjects of the Human Connectome Project Young Adult S1200 Release, the location, extent, and macroanatomical variability of the PPfpm are detailed. Demonstrating its remarkable consistency within the CS across individuals, hemispheres, handedness, and sex, the PPfpm emerges as a robust and easily identifiable structure in the human brain.

Together, this in‐depth characterization of the PPfpm provides a solid anatomical framework for future research on the relationship between the PPfpm and the functional organization of the sensorimotor hand and digit area.

## Author Contributions

A.M.M.: conceptualization, data curation, formal analysis, funding acquisition, investigation, methodology, resources, software, validation, visualization, writing – original draft, writing – review and editing. R.S.: conceptualization, funding acquisition, project administration, resources, supervision, writing – review and editing.

## Funding

The authors acknowledge funding by the International Max Planck Research School for Neurosciences at the University of Goettingen and by the German Academic Scholarship Foundation (October 2018–March 2019) to A.M.M., and by the Leibniz Association through an Outgoing Grant (LSC_OG2016_02) from the Leibniz ScienceCampus Primate Cognition (SAS‐2015‐DPZ‐LWC) to R.S.

## Ethics Statement

The authors have nothing to report.

## Conflicts of Interest

The authors declare no conflicts of interest.

## Data Availability

The structural magnetic resonance imaging data from the Human Connectome Project Young Adult S1200 Release that support the findings of this study are openly available at https://www.humanconnectome.org. Derived data are available from the corresponding author upon reasonable request.
